# Mind the gap: reconciling tropical forest carbon flux estimates from earth observation and national reporting requires transparency

**DOI:** 10.1186/s13021-023-00240-2

**Published:** 2023-11-20

**Authors:** Viola Heinrich, Jo House, David A. Gibbs, Nancy Harris, Martin Herold, Giacomo Grassi, Roberta Cantinho, Thais M. Rosan, Barbara Zimbres, Julia Z. Shimbo, Joana Melo, Tristram Hales, Stephen Sitch, Luiz E. O. C. Aragão

**Affiliations:** 1 School of Geographical Sciences, University of Bristol, Bristol, UK; 2 Faculty of Environment, Science and Economy, University of Exeter, Exeter, UK; 3 Section 1.4 Remote Sensing and Geoinformatics, Helmholtz GFZ German Research Centre of Geosciences, Telegrafenberg, Potsdam, Germany; 4 World Resources Institute, Washington, DC USA; 5 Wageningen University and Research, Wageningen, The Netherlands; 6 Joint Research Centre, European Commission, Ispra, Italy; 7 Centre for Sustainable Development (CDS), University of Brasília (UnB), Brasília, Brazil; 8 Amazon Environmental Research Institute (IPAM), Brasília, Brazil; 9 School of Earth and Environmental Sciences, Cardiff University, Cardiff, UK; 10 Earth Observation and Geoinformatics Division, National Institute for Space Research (INPE), São José Dos Campos, Brazil

**Keywords:** Forests, CO_2_ flux, LULUCF, Removal factors, Transparency, Managed land proxy, Inventories, Earth observation, Carbon budget

## Abstract

**Background:**

The application of different approaches calculating the anthropogenic carbon net flux from land, leads to estimates that vary considerably. One reason for these variations is the extent to which approaches consider forest land to be “managed” by humans, and thus contributing to the net anthropogenic flux. Global Earth Observation (EO) datasets characterising spatio-temporal changes in land cover and carbon stocks provide an independent and consistent approach to estimate forest carbon fluxes. These can be compared against results reported in National Greenhouse Gas Inventories (NGHGIs) to support accurate and timely measuring, reporting and verification (MRV). Using Brazil as a primary case study, with additional analysis in Indonesia and Malaysia, we compare a Global EO-based dataset of forest carbon fluxes to results reported in NGHGIs.

**Results:**

Between 2001 and 2020, the EO-derived estimates of all forest-related emissions and removals indicate that Brazil was a net sink of carbon (− 0.2 GtCO_2_yr^−1^), while Brazil’s NGHGI reported a net carbon source (+ 0.8 GtCO_2_yr^−1^). After adjusting the EO estimate to use the Brazilian NGHGI definition of managed forest and other assumptions used in the inventory’s methodology, the EO net flux became a source of + 0.6 GtCO_2_yr^−1^, comparable to the NGHGI. Remaining discrepancies are due largely to differing carbon removal factors and forest types applied in the two datasets. In Indonesia, the EO and NGHGI net flux estimates were similar (+ 0.6 GtCO_2_ yr^−1^), but in Malaysia, they differed in both magnitude and sign (NGHGI: -0.2 GtCO_2_ yr^−1^; Global EO: + 0.2 GtCO_2_ yr^−1^). Spatially explicit datasets on forest types were not publicly available for analysis from either NGHGI, limiting the possibility of detailed adjustments.

**Conclusions:**

By adjusting the EO dataset to improve comparability with carbon fluxes estimated for managed forests in the Brazilian NGHGI, initially diverging estimates were largely reconciled and remaining differences can be explained. Despite limited spatial data available for Indonesia and Malaysia, our comparison indicated specific aspects where differing approaches may explain divergence, including uncertainties and inaccuracies. Our study highlights the importance of enhanced transparency, as set out by the Paris Agreement, to enable alignment between different approaches for independent measuring and verification.

**Supplementary Information:**

The online version contains supplementary material available at 10.1186/s13021-023-00240-2.

## Background

### Forest carbon flux estimates in the context of the Paris Agreement

**Fig. 1:**
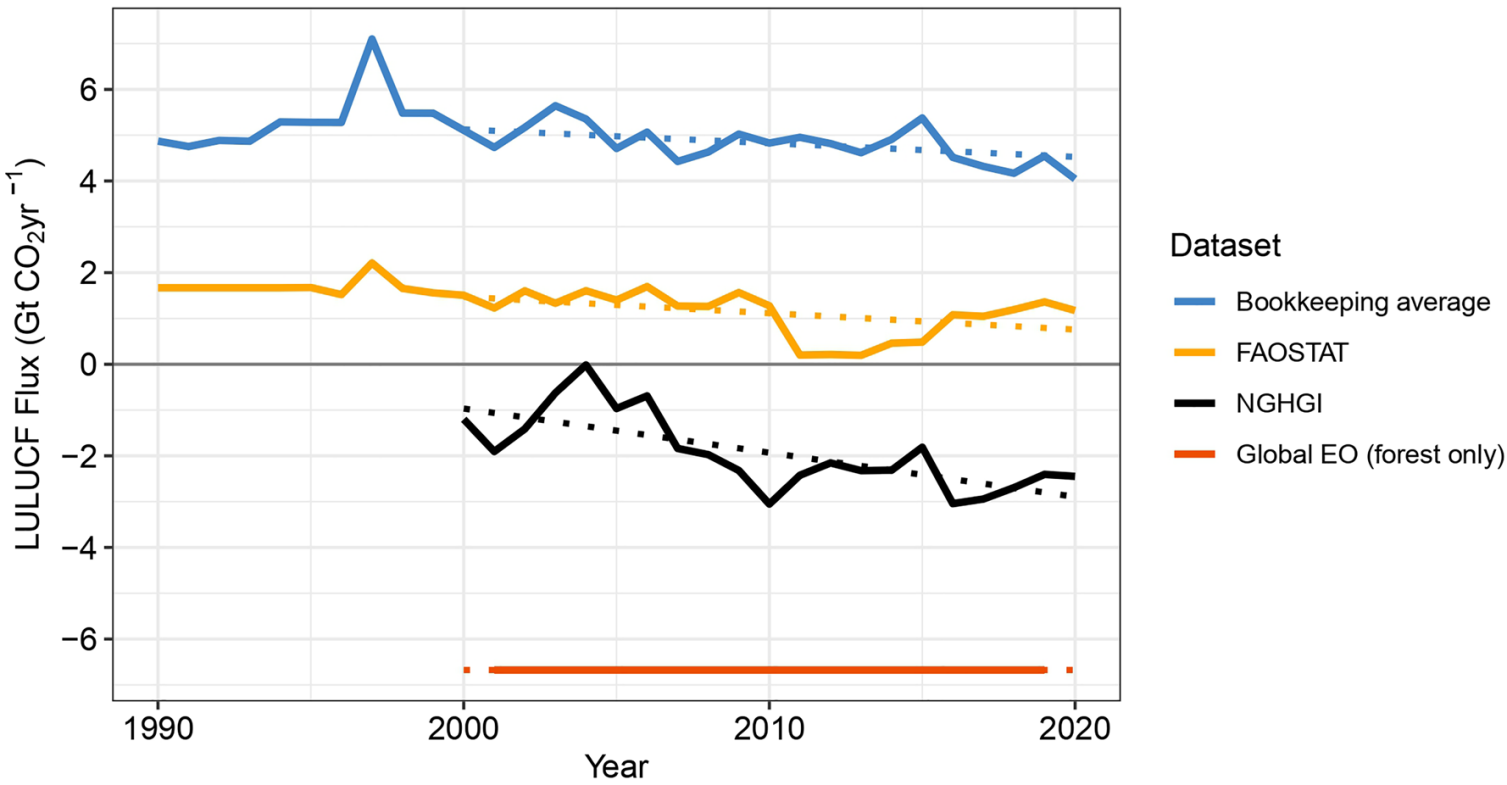
Global net CO_2_ flux due to LULUCF calculated by different datasets. Positive numbers represent a net source (emissions), negative numbers represent a net sink (removals). Light blue line: the average annual value of three bookkeeping models [3–5] as presented in [2].Yellow line: FAOSTAT includes (i) forest land converted to other land, (ii) net emissions from forest land remaining forest land, (iii) net flux from organic soils in croplands and grasslands, and from biomass burning [8, 9]. Black line: National Greenhouse Gas Inventories (NGHGI) include land-use change, and flux in managed lands [10]. Orange line: forest-only related fluxes from a Global Earth Observation (EO) dataset [17], is the sum of the gross emissions and gross removals in non-intact forests (mask: [45] (tropics); [23] (extra-tropics). Data from the Global EO were not available annually and represents the 2001 to 2019 average. The dotted lines represent the linear regression from 2000 to 2020. All trends are statistically significant (P < 0.05)

According to the Intergovernmental Panel on Climate Change (IPCC) 6th Assessment Report (AR6), the Land Use, Land-Use Change and Forestry sector (LULUCF) accounted for approximately 15% of total global net anthropogenic CO_2_ emissions between 2011 and 2020 (4.6 ± 2.0 GtCO_2_ yr^−1^) (Fig. 1) [1]. The flux, estimated from three global bookkeeping models, is due predominantly to forest-related activities such as deforestation, afforestation, reforestation, and forest management [1]. With an uncertainty of ± 50%, LULUCF is the most uncertain term in the carbon budget [2]. Prominent differences exist in both the magnitude and sign of the net anthropogenic LULUCF CO_2_ flux between global land-related datasets. Earlier studies found a large discrepancy between LULUCF estimates from the global bookkeeping models [3–5] used in the IPCC AR6 and the National Greenhouse Gas Inventories (NGHGIs) submitted to the United Nations Framework Convention on Climate Change (UNFCCC). NGHGIs are used to track progress under the Paris Agreement [6]. The latest research estimates a difference of 6.7 GtCO_2_ yr^−1^ globally for the period 2000–2020 [7]. The NGHGIs reported a small net sink (about -1.9 ± 1.0 GtCO_2_ yr^−1^ over 2000–2020) compared to the bookkeeping models reporting net emissions (about 4.8 ± 2.4 GtCO_2_ yr^−1^ over 2000–2020) (Fig. 1). In the same period, FAOSTAT reports global net land use CO_2_ emissions of 1.1 GtCO_2_ yr^−1^ (Fig. 1) [8, 9]. The difference between bookkeeping models, NGHGIs, and FAOSTAT reflects the different scopes of the country reporting to FAO, which focuses on area and biomass, and to UNFCCC, which explicitly focuses on carbon fluxes [10]. Moreover, a discrepancy of more than 5 GtCO_2_ yr^−1^ is also found between Integrated Assessment Models (IAM) and NGHGIs between 2005 and 2015 [11]. These discrepancies are problematic for the Global Stocktake (GST), as they hamper a consistent comparison between countries’ future mitigation actions, as pledged in their Nationally Determined Contribution (NDCs), and IAMs scenarios consistent with the goals of the Paris Agreement [11, 12].

Earth Observation (EO) data have been used to help understand and reconcile the gap found between datasets used in the GST at the global [11] and country scale [13]. EO data supports climate policy by providing high spatial and temporal resolution data, and are used in NGHGI reporting methods [14]. EO data can also be used as evidence to encourage more ambitious climate pledges by capturing processes currently not fully considered in NGHGIs, e.g. forest degradation [15, 16]. Currently, global models used in the GST do not explicitly use EO to quantify temporal changes in land use/cover. Recently developed global greenhouse gas (GHG) flux models based primarily on EO data, that incorporate spatially explicit, empirical observations to estimate carbon fluxes, may aid countries’ measuring, reporting and verification (MRV) of their NGHGI, and potentially provide a benchmark to evaluate the land sink from other global models [16, 17].

A recent Global EO-based dataset developed by Harris et al. [17] mapped average forest-related GHG emissions and removals (Fig. 1). The dataset provided one of the first EO integrated, globally consistent and geospatially explicit assessments of forest carbon fluxes, originally for 2001–2019 but updated through 2020. The dataset uses an inventory framework, following the IPCC guidelines and approaches used by NGHGIs. It therefore encompasses many of the same processes that NGHGIs are encouraged to consider, such as emissions and removals in all the major carbon pools in “forest land remaining forest land”, “forest land converted to other land”, and “other land converted to forest land” (see “Methodology”). Globally, the EO analysis found a large average net sink (− 6.7 GtCO_2_ yr^−1^) for 2001 to 2020, 4.8 GtCO_2_ yr^−1^ larger than that of NGHGIs. This discrepancy persisted despite adjustments made to the Global EO to exclude fluxes in primary forests/intact forests (Fig. 1), which was applied as a proxy for “unmanaged lands”, and are not considered by anthropogenically-focused NGHGIs [11]. Including EO data in the IPCC AR6 multi-data source comparison had the opposite effect from what was anticipated: rather than building consensus around a LULUCF flux estimate, the new data increased the range of reported fluxes (Fig. 1). Our study analyses the gap between NGHGIs and the EO data in specific countries, and proposes methods to reconcile and increase confidence in flux estimates from the LULUCF sector.

### Mind the gap: steps to reconciling land-use flux estimates

Studies have demonstrated that variations in land-use flux estimates are due mainly to differing definitions of what is considered to be anthropogenic fluxes on managed land [11]. Observational data alone are not able to directly distinguish between anthropogenic and non-anthropogenic fluxes as defined by IPCC (2010): (a) *direct anthropogenic effects* (e.g., deforestation and land management), (b) *indirect anthropogenic effects* (e.g. CO_2_ fertilisation, anthropogenic climate change-induced temperature changes) and (c) *natural effects* (e.g., interannual climate variability).

Anthropogenic and natural effects overlap in space and time, and it is possible only with model-based assumptions to artificially separate the effects. As a pragmatic solution to isolate anthropogenic fluxes, the IPCC Guidelines for inventory methodologies developed the “Managed Land Proxy”. This proxy defines anthropogenic Greenhouse Gas (GHG) emissions and removals by sinks (i.e. direct and in most cases indirect anthropogenic effects) as fluxes occurring on ‘managed land’, i.e. where human interventions and practices have been applied to perform production, ecological or social functions [18, 19]. Each country can define “Managed Land” in their own way, adding further complexity to comparisons between datasets. Countries’ NGHGIs collectively consider a much larger land area as “managed” compared to the assumptions applied in global models [7]. While it is “good practice” for countries to report, and spatially delineate, the area of both managed and unmanaged lands in NGHGIs, countries only need to estimate and report GHG fluxes on managed land. There is no obligation to report GHG fluxes from unmanaged lands.

Additionally, each country has different capacities, so each inventory varies in the five UNFCCC ‘principles’ for reporting NGHGIs: transparency, consistency, comparability, completeness and accuracy (Additional file 1: Table S1) [20]. Although the process of writing the IPCC guidelines is largely seen as scientific, technical and apolitical, there is “power in holding authority over how carbon is defined and managed” [21]. Quantifying the carbon fluxes of forest, a seemingly technical exercise therefore also becomes politicised. This is complicated by the fact that different forest carbon measurements produce varying results, emphasising even more the need for credible MRV and understanding the differences between these seemingly standardised, quantitative assessments [21]. Under the Paris Agreement [22], from 2024 onwards developing (“non-annex 1”) countries have stronger obligations regarding frequency, accuracy, completeness, comparability and transparency of NGHGIs. Although the Paris Agreement implementation and workflow continues to evolve and methods are constantly updated, adhering to these five principles will be increasingly important for MRV purposes, including for the voluntary cooperation between countries (Article 6.4) [22].

The main reason for the gap between NGHGIs and global non-EO models (bookkeeping and IAMs) has now broadly been explained, with approximately 80% of the discrepancy reconciled by considering the extent to which each method defines the forest sink as “managed” and thus anthropogenic [7, 11]. Other factors play a role in explaining the difference, including incomplete NGHGIs and a simplified representation of forest management in global models, but these factors likely partially counteract each other [7, 11]. Using an EO-derived map of intact and non-intact forest areas [23], Grassi et al. [7] adjusted estimates from the bookkeeping models by considering not only all direct anthropogenic effects but also indirect anthropogenic effects (identified by Dynamic Global Vegetation Models) occurring in non-intact forest areas. This adjustment expanded the definition of managed forests initially considered by the bookkeeping models to better match that of the NGHGIs.

The gap between NGHGIs and Global EO estimates is demonstrated clearly at the global scale (Fig. 1). However, reasons for the discrepancy vary by country based on the methods of each NGHGI. While a consistent global-scale analysis would be informative for the Global Stocktake, for the purposes of MRV under the UNFCCC, and the transition to the Enhanced Transparency Framework of the Paris Agreement, the discrepancies can be understood and resolved only at the country-scale [13].

Recently, numerous peer-reviewed studies and high-profile analysis by news articles have highlighted the gap between GHG flux estimates at the country-scale, mostly in the LULUCF sector [7, 24, 25]. A Washington Post article found large differences between land-based flux estimates in Malaysia’s NGHGI. Brazil is the largest contributor to LULUCF emissions (~ 25% of global LULUCF emissions) [6, 26]. However, key Brazilian biomes are also large gross carbon sinks, and Amazonia makes up about 8% of the total global land sink [2, 27]. Indonesia is the second largest contributor to LULUCF emissions, accounting for about 10% of global LULUCF emissions [2]. Brazil and Indonesia have pledged to protect and restore forests within their NDC and thus forests are critical to these countries to meet their climate targets [28].

We compared forest-related flux estimates from NGHGIs from key case study countries, Brazil, Indonesia, and Malaysia, with those from a Global EO-based analysis. Brazil’s NGHGI is regarded as one of the most complete inventories produced by Non-Annex 1 countries, with a high transparency in describing the methodology and making all datasets freely available. This made Brazil an ideal case study to compare different datasets estimating gross forest-related emissions and removals, including methodological developments for assessing the role of satellite data. For Indonesia and Malaysia, the land-cover datasets used in the NGHGIs are not publicly available for spatial analysis and download. Nevertheless, summary statistics in the NGHGIs enabled some analysis.

Using Brazil as the primary case study but also applying the same principles as far as possible to Indonesia and Malaysia this work: (i) compares estimates of forest-related GHG fluxes from a Global Earth Observation dataset and NGHGIs in three countries with large areas of tropical forest to demonstrate the utility of EO as a useful verification and evaluation tool, (ii) outlines potential reasons for differences between estimates, including the impact of different definitions of managed forest to improve the credibility of forest-related anthropogenic flux estimates, (iii) assesses implications for IPCC methods and for countries in improving their NGHGIs by using EO data when producing and improving NGHGIs, and (iv) assesses implications for future scientific research and for use of EO datasets in a policy context. Our study does not single out specific countries to scrutinise their NGHGI methods and results, but rather is designed to improve understanding of the ways in which different flux datasets can be linked and the utility of such an exercise at the country scale, within the context of the GST.

## Methodology

### Flux datasets

#### Global earth observation

The Global EO dataset used here [17] is a globally consistent framework that standardises key aspects of forest carbon inventories, including scope, definitions, assumptions and the level of transparency and completeness of the method or approach used (Table 1). The framework used the most recent IPCC guidelines for NGHGI [29] and applied NGHGI gain–loss methods to each Landsat pixel (~ 30 × 30 m) of forest (tree canopy > 30% in 2000 or subsequent tree cover gain); the framework captured transitions to and from forest, as well as forest remaining forest, but did not capture non-forest-related land uses [17]. The framework did not differentiate between managed and unmanaged forests, but the spatially explicit data allows for fluxes to be flexibly disaggregated. The framework’s initial conditions were a global map of aboveground biomass density in the year 2000 [30] in conjunction with forest extent in 2000 [31] assigned to different forest types and ages, including planted forests [32].

**Table 1:** The main driving sources in three flux datasets estimating forest-related greenhouse gas fluxes for Brazil

Driving source	Global EO	NGHGI-Brazil	SEEG-Brazil
Version/update	V1.1	NC4	9.0
time period	2001 to 2020 (whole period average)	Annually for 1990 to 2016 Manually adjusted in this study to: 2001 to 2020	1990 to 2020 (annual)
IPCC method	Gain–loss	Gain–loss* Stock-difference*	Gain–loss* Stock-difference*
Activity data			
Satellite used	– Landsat	– Landsat &Resourcesat-1	– Landsat
Main driving dataset	– Hansen et al. 1979 [31]	– RADAMBRASIL, PROBIO	– MapBiomas
Spatial resolution of land use dataset	~ 30 m	~ 30 m	~ 30 m
Emission/removal factors	Emissions: wall-to-wall aboveground biomass and soil carbon supplemented by wall-to-wall driver of loss map. Removals: various sources for different forest types	Country-specific (~ IPCC Tier 2/3 methodology) data based on field measurements and; LiDAR and peer-reviewed literature	Follows NGHGI
Forest types considered	– Primary Forest – Non-primary forest (old secondary forest) – Young secondary forest – Plantations (Forest plantations and tree crops) – (Mangroves—excluded in this analysis)	– (Non-managed forests) – Managed forest (including old growth forests under protection, conservation and indigenous lands) – Secondary forest – Plantations (forest plantations only)	– Protected forests** – Secondary forest – Plantation (Forest plantations only)
Forest transitions considered (land use and land use change)	– Forest remaining forest land – Deforestation – (Degradation)—considered retrospectively and non-spatially (Pearson et al. 2017) [72] – Afforestation and reforestation	-Forest remaining forest land – Deforestation – Selective logging (Amazonia only) – Afforestation and reforestation	– Forest remaining forest land – Deforestation – Degradation by fire – Afforestation and reforestation

The three datasets are: Global Earth Observation-based (EO) dataset [17], Brazil’s National Greenhouse Gas Inventory (NGHGI) [38] and an independent in-country inventory-style method SEEG (Sistema de Estimativa de Emissão de Gases de efeito estufa) [43]. Driving sources, as well as input datasets and assumptions made by datasets are summarised here

^*^Method used depends on transition type e.g. deforestation is stock-difference, regrowth is gain–loss

^**^ Equivalent to managed old-growth forest in the NGHGI.

Activity data were based on tree cover change [31], fires [33], and drivers of tree cover loss [34]. The Global EO dataset included emissions from all carbon pools (aboveground, belowground, dead wood, litter, soil organic carbon) and emissions from fires and peat drainage and burning. Carbon removal factors came from a variety of sources and were applied using a “stratify and multiply” approach and included carbon sequestered in aboveground and belowground biomass. In carbon accounting, the term “emission factor” can also refer to the factor applied when calculating the carbon absorbed by a system (removal from atmosphere). For clarity, here we have opted to separate the terms “emission factor” (rate at which carbon is emitted to atmosphere) and “removal factor” (rate at which carbon is removed from the atmosphere). Global geospatial data were used wherever possible (e.g. a wall-to-wall map of removal factors for young, naturally regenerating forests based on a literature review [35]), and IPCC Tier 1 default values representative for large-scale ecoregions were used otherwise (old-growth forest, old secondary forest and plantation) [29]. The use of the IPCC Tier 1 default values ensured standardisation across the regions, but may not be the most accurate estimation at local to regional scales [35–37]. In fact, for NGHGI reporting, countries are encouraged to use higher-Tier information where feasible. The final product was global maps of modelled average annual forest-related GHG emissions, removals and net flux from 2001 to 2019, but were updated to 2020 at the time of this analysis (V1.1).

#### Brazil’s National Greenhouse Gas Inventory

In the fourth National Communication (NC4) [38], Brazil’s LULUCF CO_2_ emissions and removals were available annually for 1990 to 2016 (Table 1). Brazil’s NGHGI spatially delineates which lands are unmanaged and managed and considers temporal changes in the Land Use and Land Cover (LULC) types using remote sensing data [38]. These LULC maps are available for the periods 1994 to 2002, 2002 to 2010 and 2010 to 2016, along with 2002 to 2005 and 2005 to 2010 for the Amazon biome. The LULC maps are combined with spatially explicit information on the carbon pools and associated fluxes based on field data, literature values and remote sensing. The information is used to produce matrices showing the carbon emissions and removals within managed LULC types and transitions.

Emissions and removals in non-managed lands are not reported in the flux estimates, nor are fluxes in areas that have been classified as secondary forests throughout the period of analysis areas. To use the IPCC terminology, no carbon fluxes are considered on “secondary forest lands remaining secondary forest” (Table 2). The same approach is applied to forest plantations remaining forest plantations. Land use activities occurring within these categories are assumed to be in equilibrium e.g., shifting cultivation and harvest. The key categories reported include emissions and removals in managed old-growth forests and emissions and removals in other land converted to secondary forest or forest plantations over the period analysed, i.e. very young secondary forests/plantations, for example a pasture in 2002 that was detected as secondary forest in 2010 (Table 2).

**Table 2:** Consideration of removals in different forest types by three flux datasets to estimating forest fluxes

Removals in…	IPCC category	Global EO	Brazilian NGHGI	Brazilian SEEG
Non-managed old-growth	Forest land remaining forest land	(✓)*	✗	✗
Managed old-growth	Forest land remaining forest land	✓	✓	✓
Secondary forest	Forest land remaining forest land	✓	✗	✓
Secondary forest	Other land converted to forest land	✓	✓	✓
Plantation (forest plantation)	Forest land remaining forest land	✓	✗	✗
Plantation (forest plantation)	Other land converted to forest land	✓	✓	✓
Plantation (tree crops)	Forest land remaining forest land (cropland remaining cropland)	✓	(✗)**	(✗)**
Plantation (tree crops)	Other land converted to forest land (other land converted to cropland)	✓	(✓)**	(✓)**

Forest types used in the Global Earth Observation (EO) dataset [17] and an independent estimate in Brazil—Sistema de Estimativas de Emissões e Remoções de Gases de Efeito Estufa (SEEG) have been aligned according to the IPCC LULUCF reporting categories and are compared to the Brazilian National Greenhouse Gas Inventory (NGHGI)

^*^Removals in this category are available but were not considered following a series of adjustments

^**^Removals in this category are included by the Brazilian NGHGI and SEEG but tree crops are considered in the cropland category rather than forest land

The data can be viewed in a user-friendly dashboard and are available freely to download for detailed analysis following registration (www.ccst.inpe.br/cn/), ensuring transparency. To compare the NGHGI with the Global EO estimate, we only considered CO_2_ emissions and removals occurring in forest-related categories from both datasets, namely, *“forest land remaining forest land”*, *“forest land converted to other lands”*, and *“other land converted to forest land”.* Forest classification in the NGHGI is the same used by the Food and Agriculture Organization (FAO) of the United Nations [39].

#### Other datasets: SEEG and FAOSTAT

While the primary aim of our study was to compare the GHG flux estimates from the Global EO and the NGHGI, for completeness and to aid our understanding of associated uncertainties, we also included estimates from another in-country dataset in Brazil [40] and the FAOSTAT [9].

Brazil’s System for Estimating Greenhouse Gas Emissions (Sistema de Estimativas de Emissões e Remoções de Gases de Efeito Estufa—SEEG) is semi-independent from the NGHGI and aims to produce annual estimates of GHG emissions in Brazil [40]. SEEG is updated using the latest LULC time series products from the ‘MapBiomas’ project as the input activity data to quantify the GHG flux estimates for the LULUCF sector [41] (Table 1).

The SEEG dataset follows the approach of NGHGI and only considers emissions and removals associated with anthropogenic activities, considering conservation and indigenous lands to be protected and thus managed (Table 1). A key difference between SEEG and the NGHGI is that the identification of the LULC type and annual changes in SEEG is pixel-based, as opposed to the NGHGI, which is in part based on expert visual interpretation assessment. The SEEG methodology broadly uses the same emission and removal factors as the NGHGI [42] but additionally considers fluxes in “secondary forest remaining secondary forest” (Table 2). The gross emissions and removals within the LULUCF sector can be downloaded freely from the SEEG platform and can be filtered by (i) Biome and State, (ii) Land use type, (iii) Land use change type and (iv) Land cover type (https://plataforma.seeg.eco.br/sectors/mudanca-de-uso-da-terra-e-floresta) [43]. To make the estimates from the SEEG comparable with the Global EO, we only considered CO_2_ emissions and removals occurring in forest-related categories. Previous studies have explained the differences between the NGHGI and SEEG, which include: differences in the input data, the year of land use transitions and the emission factors used [44]. The differences between these two datasets will therefore not be the focus here.

We also included estimates from FAOSTAT, which are not spatially explicit, but reported as countrywide values. FAOSTAT considers the aboveground and belowground carbon pools only, and the approach is comparable to the IPCC 2006 Tier 1 guidelines’ “stock change” approach [34]. We extracted the average forest-related CO_2_ flux for the period 2001 to 2020 by considering the categories ‘Forestland’ to represent ‘forest land remaining forest land’ and ‘Net Forest conversion’ to represent ‘forest land converted to other land’ [9].

### Estimates of GHG fluxes from different datasets

The Global EO dataset provides the emission and removals estimates as a flux per hectare in a wall-to-wall map (~ 30 m resolution). The data can be aggregated within different geographic boundaries by considering the total area of the pixels and associated fluxes within. Neither the NGHGI nor the SEEG provide spatially explicit information on the emissions and removals, but the input LULC dataset for SEEG (MapBiomas) and the NGHGI are spatially explicit. These were used to compare spatial overlap of land considered as forest in Brazil between the various approaches, specifically “managed forest” by the NGHGI (Table 1). The NGHGI managed land maps enabled us to extract the gross emissions and removals of the Global EO by forest types for greater comparability with the NGHGI. We focused on CO_2_ forest-related emissions and removals in Brazil, excluding non-CO_2_ gases.

Given the shorter period covered by the Brazilian NGHGI (2002 to 2016) (Table 1), we modified the estimates of the NGHGI to account for the temporal differences. This was important given the high emissions associated with forest loss in the early 2000s [43]. Using the annual SEEG data, we calculated the fractional difference between average gross fluxes over 2001 to 2020 (the period available for the Global EO) and 2002 to 2016 (the period available for the NGHGI). We then multiplied the fractional difference by the 2002 to 2016 NGHGI estimates, both at the biome and country scale, such that the approaches and the estimates were comparable over a common time-period (Additional file 1: Table S2).

### Adjustments to the global EO forest-related flux to make it comparable to the other approaches in Brazil

The Global EO data does not differentiate between managed and unmanaged forests. To limit the Global EO data to the managed forest fluxes reported by the NGHGI and SEEG, we analysed the impact of different adjustments to what might be considered “anthropogenic” forest-fluxes within the Global EO approach:

- No adjustment—We extracted all Global EO gross CO_2_ emissions and removals occurring within the country boundary of Brazil and compared this estimate with the NGHGI and SEEG, which consider emissions occurring on managed lands only (Table 3).
- Adjustment 1—We applied a similar approach to Grassi et al. [7]. However, instead of using an intact-forest mask, we used a primary forest mask for the year 2001 as a proxy to exclude removals in non-managed lands [45], as this dataset was already integrated within the framework of the Global EO dataset (Table 1). Additionally, we considered all CO_2_ emissions in the territorial boundary of Brazil instead of only in the non-primary forests to include post-2001 forest cover losses and associated emissions in these areas (Table 3).
- Adjustment 2a—We applied the 2016 LULC map from the NGHGI to the Global EO map considering CO_2_ emissions and removals occurring on all managed lands as defined by the NGHGI (both forest and non-forest) (Table 3).
- Adjustment 2b—We considered the *gross* CO_2_ *emissions* occurring on *all* managed lands (as Adjustment 2a). However, we only considered the *gross removals* in managed *forests* as defined by the NGHGI in 2016. The distinction allowed us to understand if there were any differences in the extent of forest land used by the NGHGI and Global EO and the impact this would have on the flux estimates (Table 3).
- Adjustment 2c—A final adjustment was made in addition to Adjustment 2b. It followed the approach used by the NGHGI of excluding removals in “secondary forest remaining secondary forest” or removals in “plantations remaining plantations” (Table 2). We excluded these categories, or those as closely matching these categories, in the SEEG and Global EO. In the SEEG dataset, we excluded removals occurring in “secondary forest remaining secondary forest”. In the Global EO, we considered old secondary forests (> 20 years old) to represent the IPCC category “secondary forest remaining secondary forest” and thus excluded removals in old secondary forest areas. We also excluded gross removals in plantation areas that did not experience any forest loss from 2001 to 2020, according to the dataset used by the Global EO [31], to represent “plantations remaining plantations”.

**Table 3:** The main adjustments made to align the Global EO forest definitions with the Brazilian National Greenhouse Gas Inventory (NGHGI)

Removals (R)/emissions (E) Flux in…	IPCC category	No adjustment	Adjustment 1	Adjustment 2a	Adjustment 2b	Adjustment 2c
Primary forest	Approx. equal to forest land remaining forest land	R: ✓ E:✓	R: ✗ E:✓	R: NA E: NA	R: NA E: NA	R: NA E: NA
Non-managed old-growth	Forest land remaining forest land	R:✓ E:✓	R:NA E: NA	R:✗ E:✗	R:✗ E:	R:✗ E:✗
Managed old-growth	Forest land remaining forest land	R:✓ E:✓	R:✓ E:✓	R:✓ E:✓	R:✓ E:✓	R:✓ E:✓
Secondary forest & plantation	Forest land remaining forest land	R:✓ E:✓	R:✓ E:✓	R:✓ E:✓	R:✓ E:✓	R:✗ E:✗
Secondary forest & plantation	Other land converted to forest land	R:✓ E:✓	R:✓ E:✓	R:✓ E:✓	R:✓ E:✓	R:✓ E:✓
Plantation (tree crops)	Cropland remaining cropland	R:✓ E:✓	R:✓ E:✓	R:✓ E:✓	R:✓ E:✓	R:✗ E:✗
Plantation (tree crops)	Other land converted to cropland	R:✓ E:✓	R:✓ E:✓	R:✓ E:✓	R:✓ E:✓	R:✗ E:✗
Other managed land	Forest land converted to other land	R:✓ E:✓	R:✓ E:✓	R:✓ E:✓	R:✗ E:✓	R:✗ E:✓

Adjustments have been broken down into gross removals (R) and gross emissions (E) in different forests type and how these are considered according to the IPCC categories. Ticks indicate that the flux was included in the adjustment, crosses indicate that the flux was excluded in the adjustment

### Evaluation of the removal factors and forest types

In addition to evaluating the discrepancy attributable to managed forest area, we considered differences in the areas of forest types and associated carbon removal factors used in each approach. We identified the removal factors used for different forest types from the spatial dataset of the NGHGI [46]. The NGHGI uses peer-reviewed data available for individual biomes [47]. The SEEG uses the same removal factors as the NGHGI and so were not included in the comparison. We calculated the modal removal factor used by the Global EO dataset for each forest type (Table 1) to be compared with the NGHGI biome-specific removal factors.

The forest types used by the NGHGI (Table 1) are available spatially explicitly within the LULC maps for available years in the NGHGI [46]. The forest types used by the SEEG are from the MapBiomas dataset [48]. MapBiomas provides pre-processed annual data on deforestation and secondary vegetation, which can be combined with their annual LULC map to obtain a forest type map in 2020. The forest types used by the Global EO were sourced from the various datasets, and included old-growth forest, old secondary forest (> 20 years old), young secondary forest (< 20 years) and plantations (Table 1) [17].

### Assessing comparability of fluxes with high uncertainty

In the tropics, the Global EO method estimated a propagated standard deviation of 45% and 110% for the gross emissions and removals, respectively [17]. The high removals uncertainty was driven by very high uncertainty in Tier 1 removal factors [17]. Brazil’s NGHGI reports an uncertainty of 32% for their estimate of LULUCF CO_2_ emissions and removals [47]. Given the high levels of uncertainty, using the traditional statistical method of determining if error bars overlap to assess the significance of the results would not be a very useful metric, as the estimates and their associated uncertainties would always overlap. Comparing the results from multiple approaches, e.g., SEEG and FAOSTAT, provides a clearer understanding of the relative uncertainty.

### Application of the analysis in other countries

A detailed analysis, like the Brazilian case study, was not possible for Indonesia and Malaysia as the LULC maps used as a basis for the NGHGIs are not publicly and readily available for download and interactive analysis from any official government website.

Indonesia’s most recent NGHGI in the third Biennial Update Report (BUR3) includes tabular annual LULUCF flux and area change data from 2000 to 2019 [49]. Data for Malaysia is from their BUR4, covering the period 1990 to 2019 [50]. Given that the Global EO is only available as an average value for 2001 to 2020, we extended the emission and removals value of both NGHGIs for 2019 to represent 2020 and then applied the 2001 to 2020 average for a more equal comparison. Key tree crops such as oil palm and acacia are considered agricultural croplands in the NGHGI of both countries; using a global plantation dataset [32], we excluded these tree crop plantations from the Global EO net flux estimate in our comparison (similar to *Adjustment 2c* made in Brazil). As far as possible, we excluded fluxes not linked to forest transitions. For the time series data in Malaysia fluxes are only reported in the main LULUCF categories, and so we assumed all emissions within the category “Settlements” were due to deforestation (Additional file 1: Table S4). The assumption results in a small higher attribution in the database compared to the inventory because there is a small contribution from the conversion of cropland to settlement that we cannot disaggregate in the time series provided by the NGHGI.

Unlike Brazil, Indonesia and Malaysia do not explicitly apply the managed land proxy, and we therefore considered all lands within their national boundaries to be managed. Thus, Global EO emissions and removals within the entire boundary of the respective countries were considered in comparison with the NGHGI. Given the importance of peat fire and peat decomposition emissions in these two countries, which include non-CO_2_ emissions, for Indonesia and Malaysia we considered all GHG emissions from all flux datasets. Both Indonesia [49] and Malaysia [50] report emissions and removals in key IPCC categories similar to those outlined in Table 2. The stated uncertainties of Indonesian and Malaysian NGHGIs are about 14% and 15%, respectively, when excluding the LULUCF sector. These values increase to 20% and 57% for Indonesia and Malaysia, respectively when LULUCF is included. LULUCF is therefore a key source of uncertainty in these two countries [49, 50].

## Results

### Making the extent of managed land more comparable for flux estimates in Brazil (Adjustments 1, 2a, 2b)

**Fig. 2:**
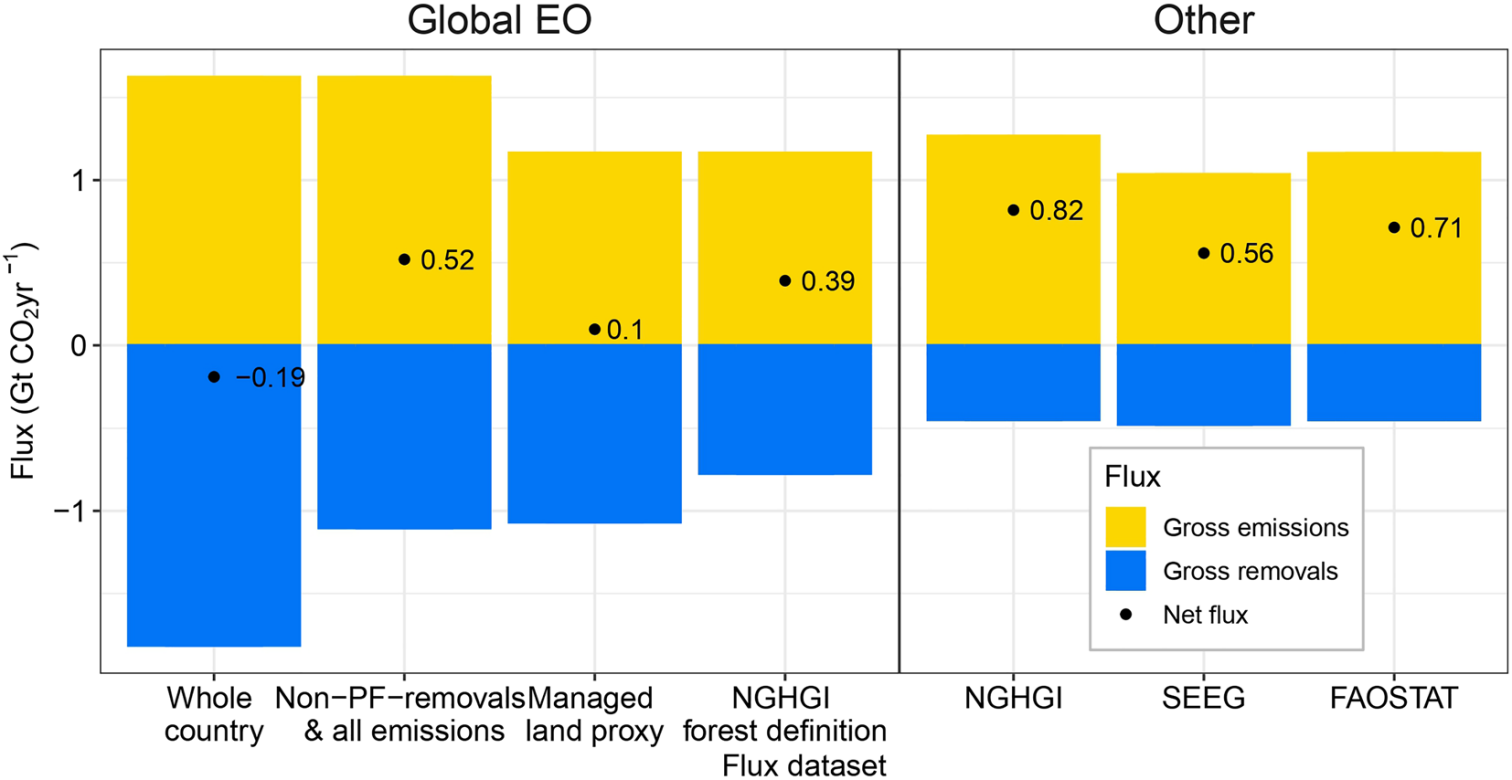
Adjustments to the Global Earth Observation (EO) forest-flux estimate to increase comparability with other datasets for Brazil. Bars denote the average annual gross emissions/removals and black points and associated text denote the net forest carbon fluxes over the period 2001 to 2020. The left panel shows the impact of adjustments made to the Global EO dataset [17] when considering managed forest/land to align with the definitional approach of other datasets. Non-PF refers to Non-Primary Forest. Right panel shows the other flux datasets, namely the National Greenhouse Gas Inventory (NGHGI) of Brazil [38], SEEG-Brazil [43] and FAOSTAT [9] for Brazil. Note the original time-period for NGHGI was 2002 to 2016, and values have been adjusted to reflect the period 2001 to 2020 (see “Methodology”). Uncertainty measures have been excluded from the figure for clarity due to the high uncertainty associated with all flux datasets (see “Methodology”)

Assuming all forest gross emissions and removals within the country boundary of Brazil were considered as managed (*No adjustment*), the Global EO net flux would be − 0.2 GtCO_2_ yr^−1^, a small sink from 2001 to 2020 (Fig. 2: 1st bar). Over the same period, the other flux datasets, the NGHGI, SEEG and FAOSTAT, report a net source of 0.8 GtCO_2_ yr^−1^, 0.6 GtCO_2_ yr^−1^, and 0.7 GtCO_2_ yr^−1^, respectively (Fig. 2: bars 5 to 7). We adjusted the extent of the managed land definition in the Global EO dataset to make it more comparable with the NGHGI and other flux datasets, and recalculated the forest flux (Fig. 2).

**Fig. 3:**
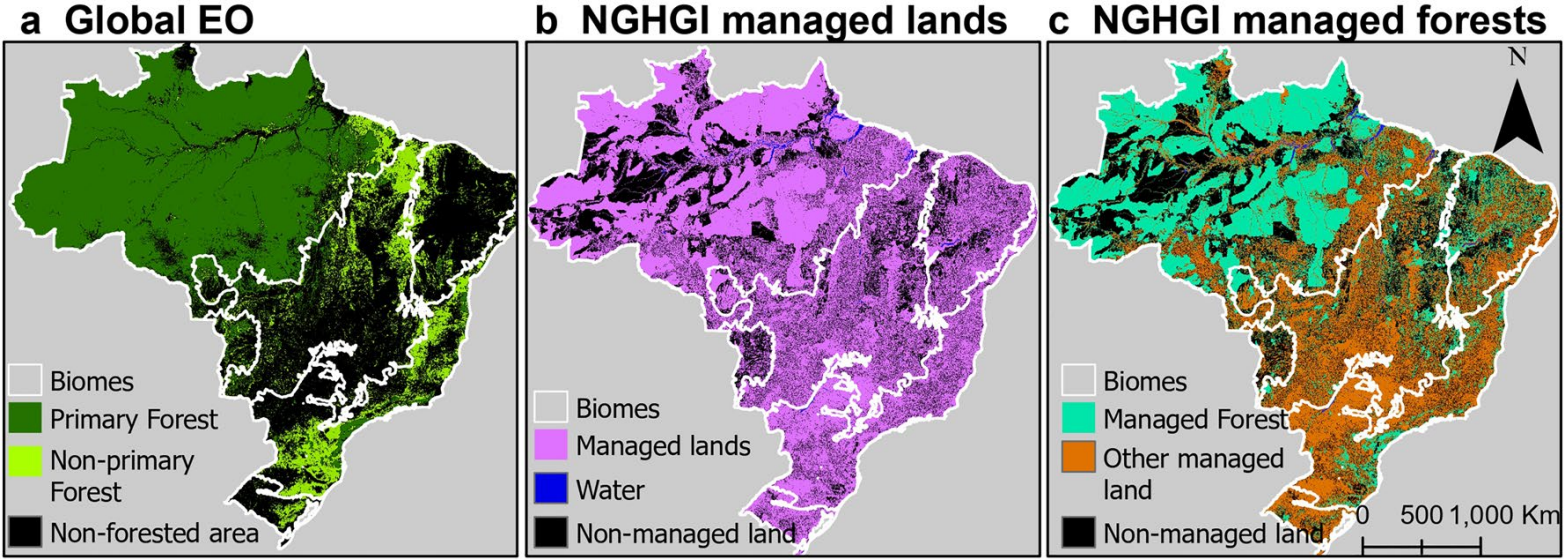
Land cover maps showing the extent of managed/unmanaged lands according to definitions of different datasets. The datasets are a Global Earth Observation (EO) dataset and the Brazilian National Greenhouse Gas Inventory (NGHGI). **a** is the extent of primary (~ old growth) forest and non-primary forest used by the Global EO dataset [17]; **b** the regions classed as managed and non-managed areas according to the Brazilian NGHGI in 2016 [38]; and **c** are the same regions seen in **b** of managed land split up according to managed forest and other managed lands

When considering gross emissions on all lands in the country boundary and only removals occurring in non-primary forest lands in the Global EO dataset (*Adjustment 1*) (Fig. 3a), the forests become a net source of 0.5 GtCO_2_ yr^−1^ (Fig. 2: 2nd bar). While the Global EO net flux is only about a third lower than the NGHGI, the gross emissions and removals both remain higher. The differences likely arise because the spatial extent of these forest types is not similar in Brazil (Fig. 3a and c). Spatially, the non-primary forest lands only overlap with 11% of managed forests in the NGHGI (Additional file 1: Table S3). When aggregated, the total *“non-primary forest”* area (507 Mha) is very similar to the NGHGI “managed forest” area (397 to 484 Mha), emphasising the importance of spatial analysis when considering proxies for managed forests (Additional file 1: Figure S1).

We applied the NGHGI spatial mask of all managed land (Fig. 3b) to the Global EO flux dataset, which yielded a net forest flux of 0.1 GtCO_2_ yr^−1^, making it a small source (Fig. 2: 3rd bar) (*Adjustment 2a*). Gross emissions are very similar to other datasets, with the estimate from the Global EO being 0.1 GtCO_2_ yr^−1^ smaller compared to the NGHGI. Gross removals remain ~ 0.6 GtCO_2_ yr^−1^ higher than the NGHGI. This suggests that deforestation areas and emissions factors are similar in Brazil’s NGHGI and the Global EO under this adjustment, and that most of the difference is due to differences in forest classification and removal factors applied in managed forests.

With an additional Global EO data adjustment to consider removals occurring only within “managed forest” as defined by the NGHGI but still include emissions from all managed land (Fig. 3c), the net flux was a source of 0.4 GtCO_2_ yr^−1^ (Fig. 2: 4th bar) (*Adjustment 2b*). Despite the same definition of managed forest, the gross removals flux of the Global EO data is two fifths larger than the NGHGI.

**Fig. 4:**
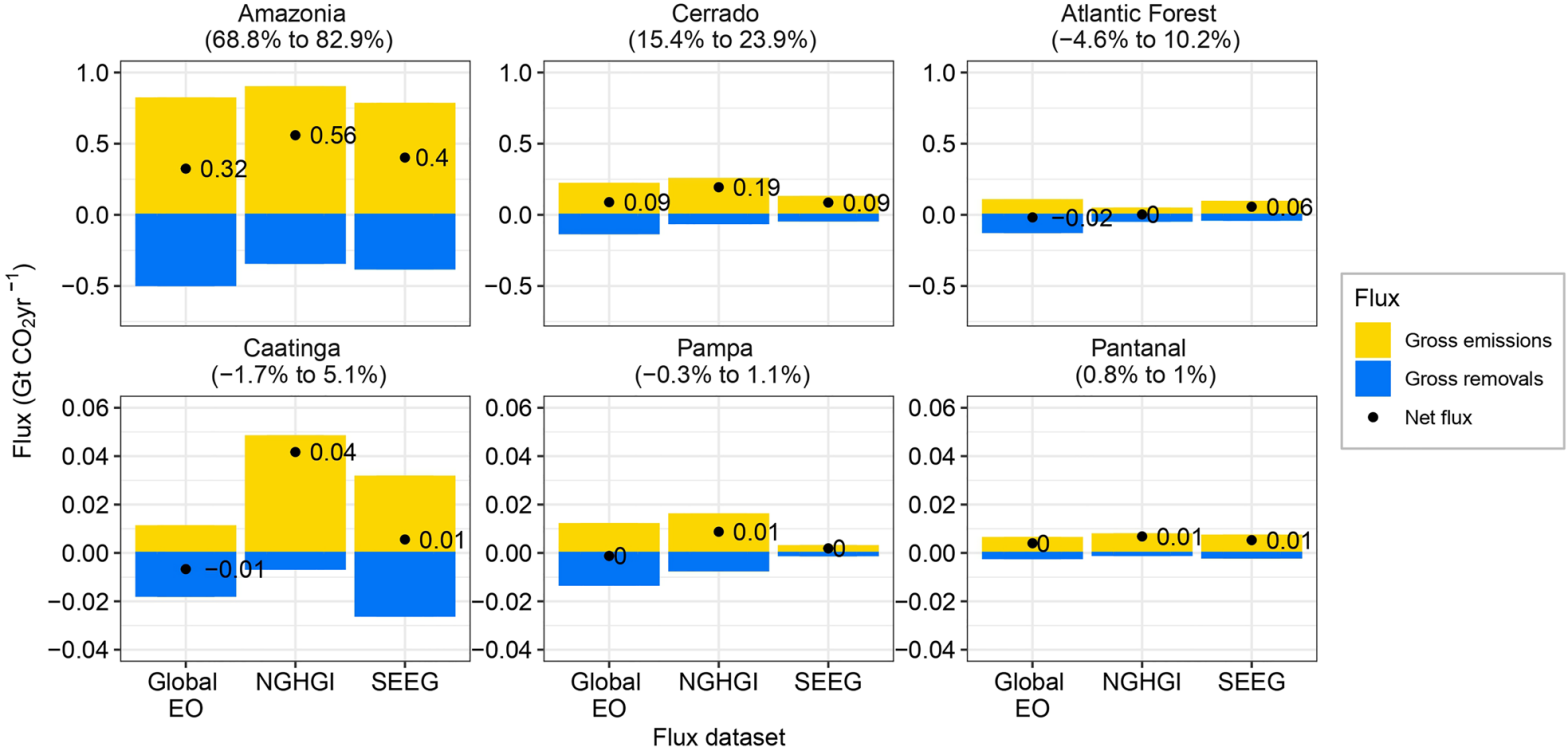
Average forest carbon flux estimates from different datasets over the period 2001 to 2020 across the Brazilian biomes. Bars denote the average annual gross emissions/removals and black points and associated text denote the net forest carbon fluxes over the period 2001 to 2020. All methods only consider removals in managed forest lands. The Global Earth Observation (EO) [17] values are based on the *Adjustment 2b*—i.e. using the NGHGI managed forest lands [38] to extract area and removals. Black points are the net flux over the period of analysis. Note the original period for NGHGI was 2002 to 2016, and values have been adjusted to reflect the period 2001 to 2020 (see “Methodology”). Percentages in brackets above each panel represents the percentage contribution of each biome to Brazil’s total net flux over the period. The range represents the lower and upper contribution amongst the three presented methods. Note varying scales on Y-axis

When we disaggregated *Adjustment 2b* at the biome scale, differences between the flux datasets became more apparent (Fig. 4). In Amazonia, the biome with the biggest contribution to the net flux (69% to 83%), the gross emissions in the Global EO (0.8 GtCO_2_ yr^−1^) are very similar to the NGHGI (0.9 GtCO_2_ yr^−1^). However, gross removals are 1.5 times lower in the NGHGI. In the Cerrado and Atlantic Forest, which together make up approximately 11% to 34% of the net flux, gross removals are up to two and three times higher, respectively, in the Global EO compared to the NGHGI (Fig. 4). Given that these comparisons only included gross removals in managed forests for the Global EO data, we could not reconcile the remaining differences observed at the biome and country scale by only considering the extent of forest cover and whether it is managed forest or not (Figs. 2 and 4).

**Fig. 5:**
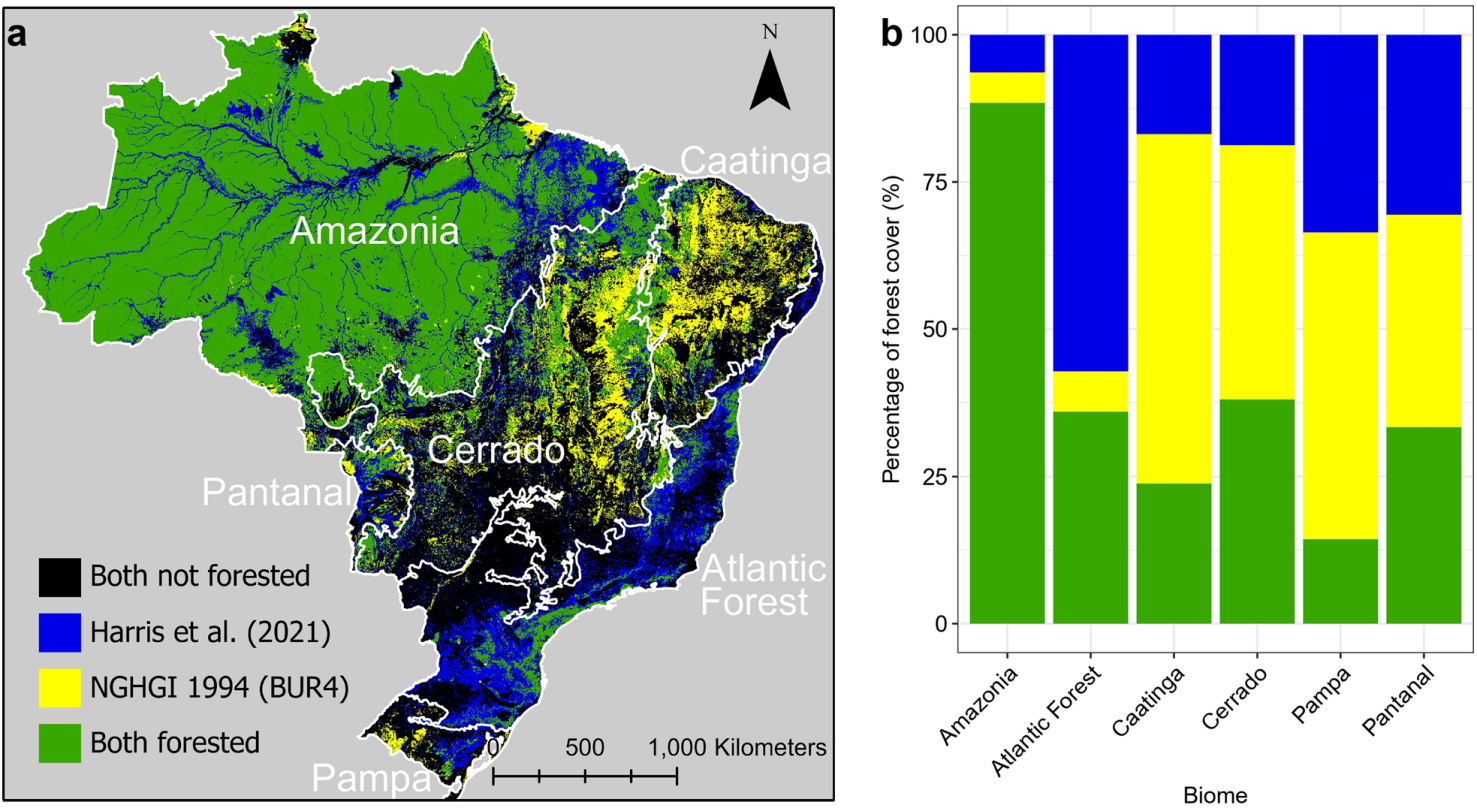
Forest cover extent according to the Global EO dataset and Brazil’s NGHGI. Data is shown spatially (**a**) and aggregated by biome (**b**). The forest cover relates to the year 2000 for the Global EO [17] and 1994 for NGHGI dataset [38]. The year 1994 for the NGHGI was chosen as the data is not available annually. The Y axis in **b** represents the maximum forest coverage by combining both datasets, removing areas considered non-forested areas in both datasets

We found considerable spatial differences when comparing the extent of forest cover in the Global EO and NGHGI datasets (Fig. 5a, b). The largest differences are outside of the humid forest-dominated Amazon biome. In the Atlantic Forest, the other key humid forest biome, only 35% of the total potential forest area (the areas considered as forest in either or both datasets) was classified as forest cover in both datasets, providing some understanding of the observed flux differences. Across the other four biomes, which are not dominated by humid-forest cover, the area considered to be forest is higher in the NGHGI. These regions were not considered forest in the Global EO study as their tree canopy cover per pixel was less than 30%. The NGHGI does not define such a threshold and applies the FAO classification [38].

### Relative contribution of different forest types to gross removals in Brazil

Following Adjustments 2a and 2b, we found that most of the discrepancy between forest fluxes was in the gross removals component. To explore potential reasons for this discrepancy, we disaggregated the removals flux according to forest types included in each flux dataset and the area occupied by each forest type in 2020 (Table 4). The difference in area occupied versus relative gross removals of respective forest types also varies across the six biomes (Additional file 1: Figures S3 and S4). The reasons for differences in both area and gross removals are interconnected and difficult to untangle.

**Table 4:** The 2020 area of different forest types and associated carbon removal flux according to different datasets in Brazil

Forest type	Global EO***		NGHGI-Brazil		SEEG-Brazil	
	Area (2020) (1000 ha)	Gross removals (GtCO_2_ yr^−1^)	Area (~ 2020)* (1000 ha)	Gross removals* (GtCO_2_ yr^−1^)	Area (2020) (1000 Mha)	Gross removals (GtCO_2_ yr^−1^)
Managed old-growth	195,954	− 0.42	220,158	− 0.32	217,702	− 0.31
Secondary forest	23,998	− 0.16	21,877**	− 0.05**	8547	− 0.16
Plantation	6779	− 0.19	11,144	− 0.08	6311	− 0.003
Other land	31,732	–	5284	–	25,903	–
Total	258,463	− 0.77	258,463	− 0.45	258,463	− 0.48

The carbon removal component is for the average annual removal flux for the period 2001 to 2020. For an explanation of different data sources see “Methodology”. For the National Greenhouse Gas Inventory (NGHGI) [38] only pixels in the managed forest area outlined in NC4 were used (green regions in Fig. 3c)

^*^As the NGHGI is only available up to 2016, numbers for the NGHGI have been adjusted by multiplying by the fractional difference in the area/gross removals of each forest type in 2016 and 2020 according to SEEG (MapBiomas) SEEG-Brazil [43]

^**^This value also includes selective logging areas and gross removals from Amazonia

^***^These values are based on *Adjustment 2b*—i.e., using the NGHGI managed forest lands to extract area and removals in the Global Earth Observation (EO) [17]

According to the NGHGI, plantations occupy an area of approximately 11 Mha and have a gross removals flux of − 0.08 GtCO_2_ yr^−1^. According to the Global EO estimate, plantations occupy about two-thirds of this area (6.8 Mha) but estimated removals in plantations are more than double compared to the NGHGI estimate (− 0.19 GtCO_2_ yr^−1^) (Table 4). This can partly be explained by the fact that the NGHGI and SEEG do not consider removals in “plantations remaining plantations” (Table 2). Furthermore, tree plantations such as rubber, acacia and oil palm are included within forest flux estimates in the Global EO dataset, whereas, in the NGHGI and SEEG, they are considered as cropland and are, therefore, not included in forest-related removals (Table 2). Given that we adjusted the Global EO gross removals to consider only “managed forest” areas according to the NGHGI (*Adjustment 2b*), the relative contribution to both the area and gross removals of tree crops to the final adjusted Global EO is small (Additional file 1: Figure S2).

The Secondary Forest area in the SEEG estimate (8547 Mha) is two-thirds smaller than the NGHGI (21,877 Mha), yet gross removals are three times higher in SEEG (− 0.16 GtCO_2_ yr^−1^), compared to the NGHGI (− 0.05 GtCO_2_ yr^−1^). The difference can partly be explained by the fact that the SEEG considers removals in “secondary forest remaining secondary forest” but the NGHGI does not (Table 2). Secondary forest removals in the Global EO are also three times higher than in the NGHGI, despite occupying a similar total area (Table 4). Across the three datasets, the area of old-growth forest is approximately the same (~ 20 Mha), and the gross removals are a third larger in the Global EO (− 0.4 GtCO_2_ yr^−1^) compared to the NGHGI and SEEG (~ 0.3 GtCO_2_ yr^−1^) (Table 4), but within the uncertainty of the NGHGI dataset (32%—see “Methodology”).

### Reconciling the difference in gross removals between flux datasets in Brazil (Adjustment 2c)

**Fig. 6:**
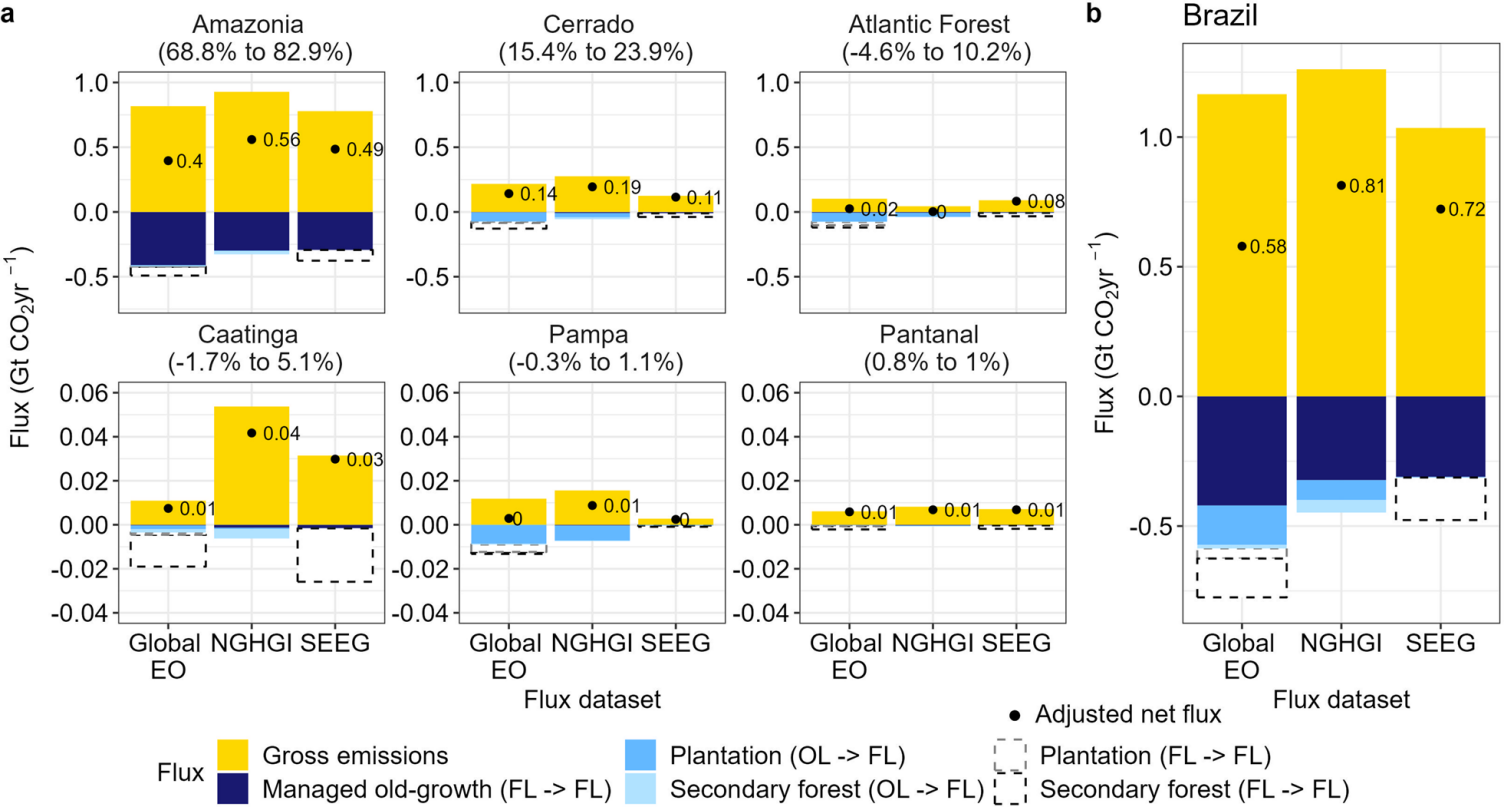
Adjusted comparison of the average annual forest carbon fluxes between different flux datasets for 2001 to 2020. Panels are split up across the six biomes of Brazil (**a**) and across the whole country (**b**). Bars denote the average annual gross emissions/removals and black points and associated text denote the net forest carbon fluxes. The three flux datasets are the Global Earth Observation (EO) [17], the Brazilian National Greenhouse Gas Inventory (NGHGI) [38] and the independent estimate (SEEG) [43]. Adjustments to the Global EO data exclude gross removals in ‘Forest Land remaining Forest Land’ (FL → FL) in plantations and secondary forest (dashed areas) in the net flux calculation. The net flux is therefore only comprised of: FL → FL in managed old-growth forests; Other Land converted to Forest Land (OL → FL), namely secondary forest and Plantation areas. All datasets either only consider removals in managed forest lands or have been adjusted such that only these areas are considered. Black text refers to the adjusted net flux over the period of analysis. Note the original time-period for NGHGI was 2002 to 2016, and values have been adjusted to reflect the period 2001 to 2020 (see “Methodology”). The values for the Global EO are based on *Adjustments 2a to 2c*—i.e., using the NGHGI managed forest lands to extract area and removals in the Global EO and then excluding the aforenamed categories (see “Methodology”). Note the different scales in the Y-axis

We made a final adjustment to the Global EO data (*Adjustment 2c*) by excluding older plantation and secondary forest areas (Fig. 6). This adjustment accounted for the fact that the NGHGI treats fluxes within these categories as net zero (see “Methodology”, Table 2). Considering this final adjustment, the difference between the NGHGI and Global EO/SEEG is halved compared to *Adjustment 2b*. The NGHGI net flux remains higher (0.81 GtCO_2_ yr^−1^) but only by 28% and 10% for the Global EO (0.58 GtCO_2_ yr^−1^) and SEEG (0.72 GtCO_2_ yr^−1^), respectively (Fig. 6), the smallest net difference compared to any of the previous adjustments applied (Fig. 2).

### Explaining the remaining discrepancy: removal factors and forest type

There are a few reasons why a gap between the Global EO and other flux datasets remains after *Adjustment 2c* despite considering the same areas of managed forests and associated fluxes; each flux dataset used different removal factors and spatial extents of forest type. By comparing the removal factors and forest types used in the various datasets, we can explore the remaining discrepancies without changing the methodology used by the flux datasets.

Looking at the differences in removal factors used for old-growth forests across the Brazilian biomes, which make up 55–70% of the gross removals flux (Table 4), we see that IPCC Tier 1 removal factors used by Global EO dataset are larger compared to the NGHGI (Table 5). In Amazonia, the removal factor used by the Global EO is a fifth higher than the NGHGI removal factor (Table 5). This helps to explain the higher gross removals in this forest type across the country (Table 4) and regionally, e.g., in Amazonia (Additional file 1: Figure S4). In the other biomes, the removal factors are higher, by between 20–850% (Table 5). However, the relative contribution of old-growth forest carbon removal to the total gross removals is considerably less than Amazonia (Additional file 1: Figure S4). The results highlight the impact of using more region-specific, or “higher-Tier” emission/removal factors to improve assessment accuracy.

**Table 5:** Old-growth Forest removal factors used by different flux datasets and disaggregated by biomes in Brazil

Biome	NGHGI and SEEG (Mg C ha^−1^ yr^−1^)	Global EO (Mg C ha^−1^ yr^−1^)	Percentage difference (global EO/NGHGI) (%)
Amazonia	0.48	0.59 (0.24)	+ 23
Atlantic Forest	0.44	0.59 (0.24)	+ 34
Cerrado	0.2	0.24 (0.59)	+ 20
Pantanal	0.2	0.24	+ 20
Pampa	0.44	NA (0.59 = Old SF)	+ 34
Caatinga	0.1	0.95	+ 850

For all datasets, values include above and below ground carbon. The National Greenhouse Gas Inventory (NGHGI) and SEEG) [38] applied a single, biome-specific, removal factor for each biome. The calculated modal removal factor in each biome for the Global Earth Observation (EO) dataset [17] (see “Methodology”) is shown. Values in brackets indicate old-growth removal factors also used but where the associated ecozone did not account for a large area within the biome. In the Pampa biome the Global EO did not detect any old-growth (primary forest) areas and only old secondary forest (Old SF) regions were identified

The removal factors in forest plantations are generally larger in the Global EO method, compared to the NGHGI, with a range of 9.5% to 59% (Table 6). In young secondary forests, those regrowing for less than 20 years, the Global EO average removal factors are also generally larger, between 29 and 543% (Table 6).

**Table 6:** Plantation and Secondary Forest removal factors used by different flux datasets and disaggregated by Brazilian biomes

Biome	NGHGI (Mg C ha^−1^ yr^−1^)	Global EO (Mg C ha^−1^ yr^−1^)	Percentage difference (Global EO/NGHGI)
Plantations			
Amazonia	12.5 (8.3 to 12.66)	11.2 (4.72 to 20.3)	− 10.4%
Atlantic forest	11.6 (10.5 to 12.6)	12.7 (4.72 to 20.3)	+ 9.5%
Cerrado	12.6 (11.1 to: 12.6)	16.6 (4.72 to 20.3)	+ 31.7%
Pantanal	12.7 (12.7 to 12.7)	20.2 (4.72 to 20.3)	+ 59.1%
Pampa	11.0 (11.0 to 11.0)	12.9 (4.72 to 20.3)	+ 17.3%
Caatinga	12.6 (12.1 to 12.7)	17.0 (4.72 to 20.3)	+ 34.9%
Young secondary forests (< 20 years)			
Amazonia	3.1 (0.6 to 5.2)	6.4 (3.9 to 8.0)	+ 106%
Atlantic forest	1.7 (1.7 to 1.7)	4.5 (2.0 to 7.6)	+ 165%
Cerrado	2.7 (0.6 to 4.7)	4.3 (2.5 to 7.3)	+ 59%
Pantanal	2.8 (0.6 to 4.7)	3.6 (2.3 to 5.5)	+ 29%
Pampa	3.2 (0.6 to 4.7)	2.8 (1.6 to 5.1)	− 13%
Caatinga	0.7 (0.6 to 1.0)	4.5 (2.5 to 7.2)	+ 543%
Old secondary forests (> 20 years)			
Amazonia	–	1.36 (1.60)	–
Atlantic forest	–	1.36 (1.60)	–
Cerrado	–	1.60 (1.36)	–
Pantanal	–	1.60	–
Pampa	–	0.59	–
Caatinga	–	1.60	–

For all datasets, values include above and below ground carbon. Secondary forests are further disaggregated by forest-age classes. This shows the average removal value for each biome, which in the National Greenhouse Gas Inventory (NGHGI) [38] varies according to the type of land use transition taking place, and in the Global Earth Observation (EO) [17] varies depending on the type of forest plantation. The calculated modal removal factor in each biome for the Global EO dataset (see “Methodology”) is shown. Values in brackets indicate removal factors also used but where the associated ecozone did not account for a large area within the biome

As the NGHGI does not include removals in ‘secondary forest remaining secondary forest’, it only uses removal factors applicable for younger (< 20 years) secondary forest (‘other land converted to forest land’). The Global EO product does, however, distinguish between old secondary and young secondary forests. All forested regions not identified as primary forests, having tree cover gain, plantations, or mangroves in 2000 are classified as ‘old secondary forests’ (> 20 years). The appropriate removal factors are then used according to the updated IPCC guidelines [42]. The average removal factors of old secondary forest in the Global EO are lower than the NGHGI young secondary forests removal factors, but higher than old-growth forests ones (Table 6).

**Fig. 7:**
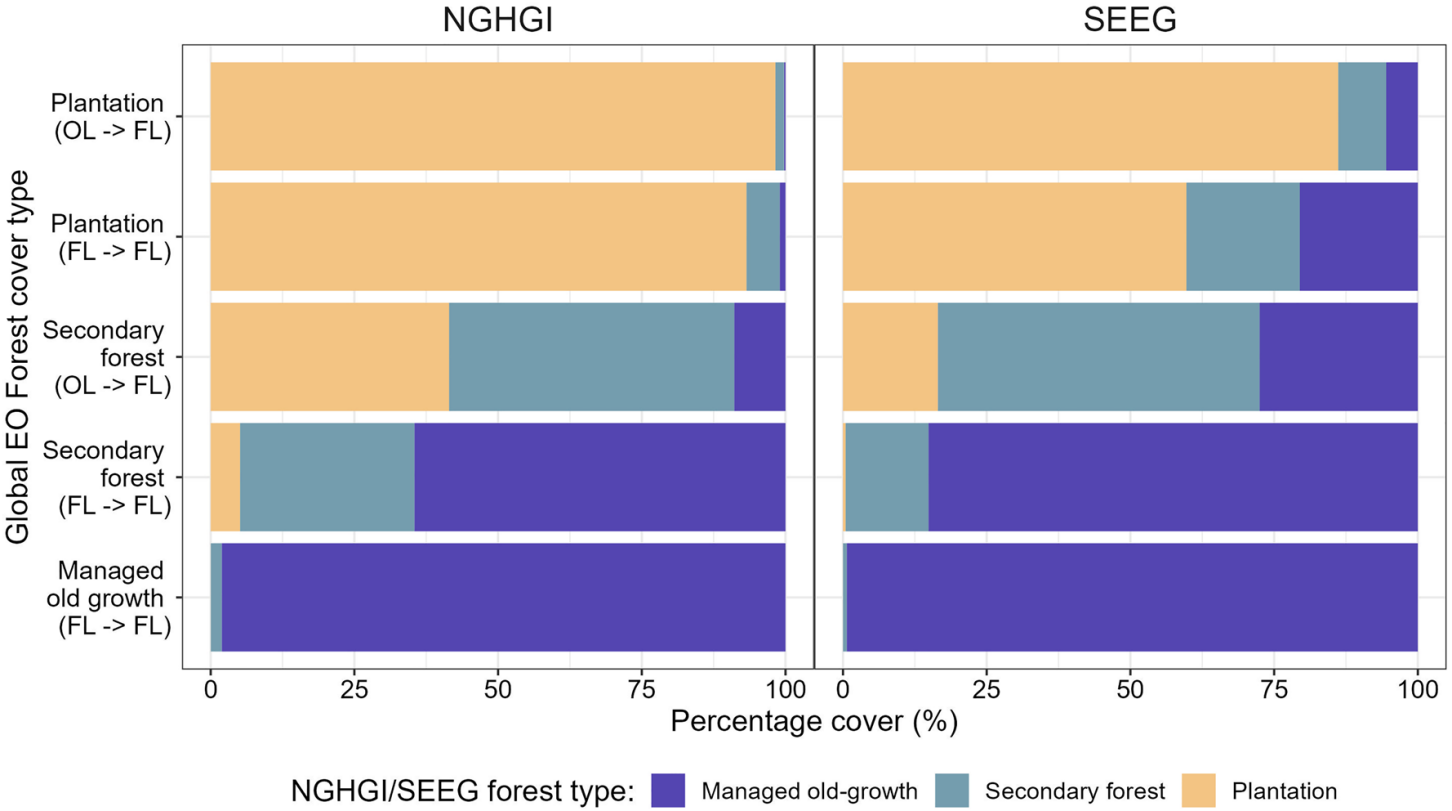
Percentage of pixels classified as different forest sub-types in Brazilian datasets compared to the Global Earth Observation (EO) dataset. The two national datasets are the National Greenhouse Gas Inventory (NGHGI) and the SEEG. Each bar represents a forest cover type as identified by the Global EO dataset in 2020 [17]. The colours within each bar represent the forest type as identified by the NGHGI in 2016 (left panel) [38] and the SEEG in 2020 (right panel) [43]. The forest cover types of the Global EO have been adjusted to match the IPCC reporting categories, where FL → FL refers to ‘Forest Land remaining Forest Land’, and OL → FL refers to ‘Other land converted to Forest Land’

The other remaining discrepancy, related to the spatial extent of forest types across the three flux datasets, will also dictate the type of removal factors applied. At the country-scale, we quantified differences in the classification of forest type between the datasets (Fig. 7). We found the most noticeable difference in the classification of managed old-growth forests in the NGHGI and SEEG and ‘secondary forest remaining secondary forest’ in the Global EO. Here, 65% and 85% of the pixels classified as Secondary Forest (Forest land remaining forest land, FL → FL) by the Global EO method were classified as managed old-growth forest by the NGHGI and the SEEG, respectively (Fig. 7). Of the forest-cover types, the classification of managed old-growth (FL → FL), plantations remaining plantations (FL → FL) and other land converted to forest land (OL → FL) were the most consistent across the three datasets, with up to 98% consistency between the Global EO and the NGHGI.

The differences in removal factors and forest types used by each flux dataset highlights the individual approaches, boundary conditions and methodological priorities. We therefore could not reconcile the flux further without entirely re-doing the work of each separate flux dataset. The remaining differences in the flux between the Global EO (0.58 GtCO_2_ yr^−1^), NGHGI (0.81 GtCO_2_ yr^−1^) and SEEG (0.72 GtCO_2_ yr^−1^) (Fig. 6) give a measure of the uncertainty of gross and net flux estimates for forests in Brazil.

### Differences in the key carbon pools with implications for gross emissions in Brazil

Differences in the major carbon pools and how the various datasets handle deforestation and degradation will influence the emission factors and help to explain some of the observed differences in gross emissions.

The Brazilian NGHGI, SEEG and Global EO consider all five carbon pools: aboveground carbon, belowground carbon, dead wood, litter, and soil organic carbon, but apply different assumptions. The Global EO applies IPCC default ratios to estimate belowground carbon from aboveground carbon, climate-based ratios to estimate deadwood and litter carbon from aboveground carbon, and a global map of soil organic carbon in mineral soils. Where available, the NGHGI uses biome specific conversion ratios for the belowground carbon and specific values for deadwood and litter and otherwise uses IPCC default values [38].

**Fig. 8:**
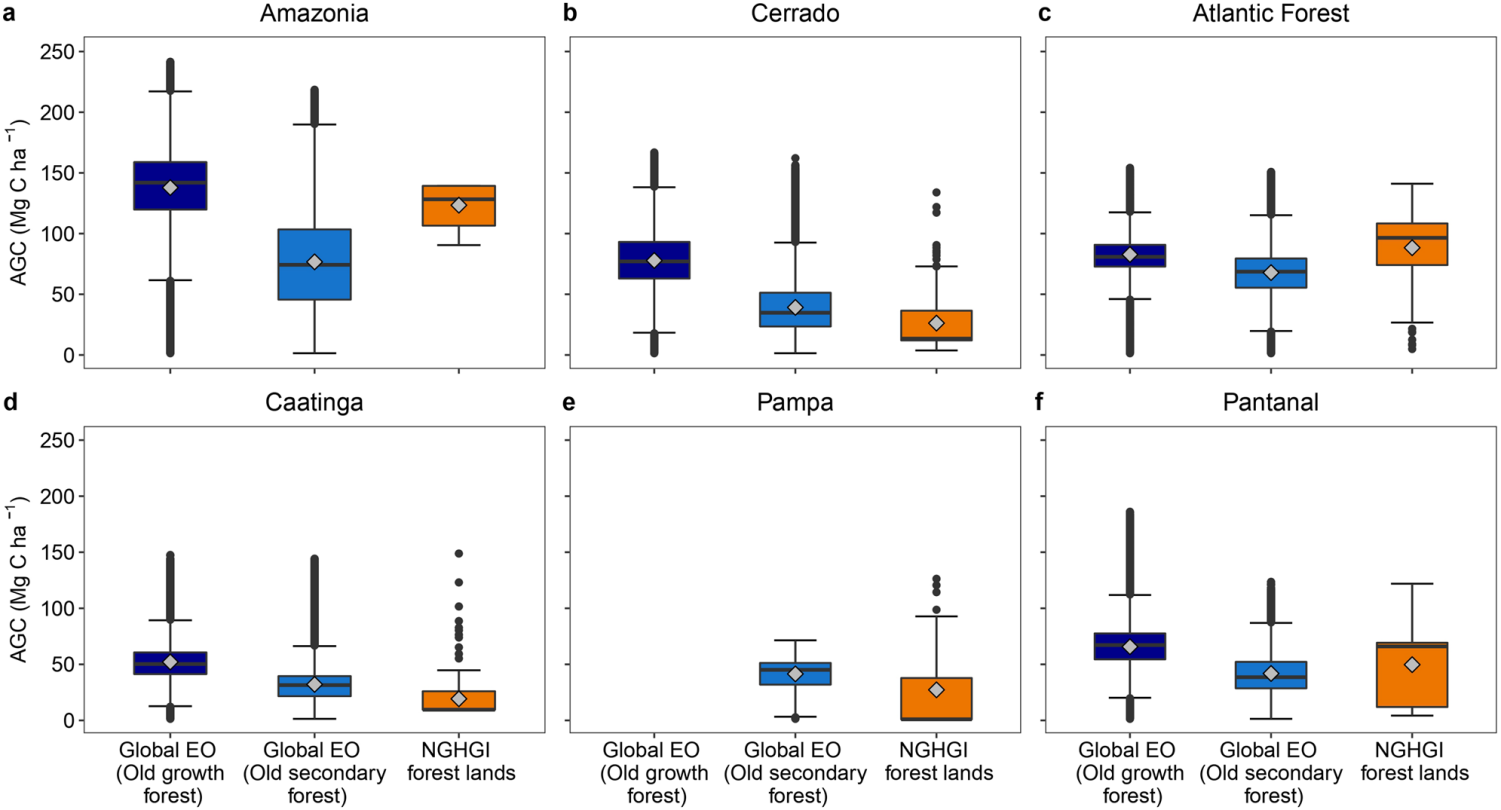
Aboveground Carbon (AGC) estimates according to forest types used within different datasets in Brazil’s biomes (**a** to **f**). The datasets are the Global Earth Observation (EO) dataset [17] and the National Greenhouse Gas inventory (NGHGI) [38]. Box plots show the range of the AGC values for regions identified as old growth forests and old secondary forests by the Global EO for the year 2000. Values for the NGHGI relate to AGC prescribed to forest pixels or former-forested pixels. Regions of old secondary forests in the Global EO relate to only regions that were classed as managed old-growth forest by the NGHGI for a more consistent comparison. Grey diamonds denote the mean AGC value

For estimating aboveground carbon (AGC) densities, the Global EO uses a pixel-based, remote sensing approach [51] and hence the spatial variability of AGC estimates is greater compared to the NGHGI (Fig. 8). The NGHGI prioritises using structural field-derived data from various in-country inventories across the six biomes, literature derived estimates, and for the Amazon used LiDAR as well [47]. In both Amazonia and the Atlantic Forest, the mean old-growth forest AGC densities for the Global EO are similar to the NGHGI (Fig. 8), which may partly be because both flux datasets rely on field-based calibrations applying similar allometric equations. The estimates diverge more in the other biomes; however, the interquartile ranges (IQR) generally overlap.

There are differences in AGC densities between the Global EO classification of old-growth forests and old secondary forests. In Amazonia and Cerrado, the IQR of the old-growth and old secondary forests do not overlap, suggesting differences in the forest aboveground carbon dynamics between these two classifications. As the NGHGI does not distinguish between old-growth forests and old secondary forests [47], it is not clear which forest types the AGC estimates encompass (Fig. 8).

The method to determine deforestation and degradation varies across the flux datasets and influences the gross emissions. The Global EO uses a remotely sensed dataset to identify forest cover loss and includes losses associated with stand-replacing disturbances such as fire and logging. Smaller scale degradation events (< 30 m) may go undetected and, therefore, unquantified by the medium resolution satellite observations [17]. The representation of degradation in the Global EO dataset is, therefore, incomplete. The NGHGI only considers degradation via selective logging, and only in Amazonia, thus potentially explaining why the Global EO estimate for gross emissions is lower than the NGHGI. The SEEG does provide an estimate of emissions by fire unrelated to deforestation in all biomes, however these are currently an additional dataset, and are not included in this analysis [42].

### Comparing flux estimates of different datasets in South-East Asia

The net flux and associated gross emissions and removals for Indonesia are remarkably similar in the Global EO (0.55 GtCO2e yr^−1^) and the NGHGI (0.57 GtCO2e yr^−1^) dataset [17]. Like the NGHGI, the Global EO study also includes emissions from peat fires and peat decomposition, key emission sources in Indonesia, although the way those are calculated differ between the two datasets. The relative contribution of different sources (e.g. deforestation, peat drainage and peat fires) to the gross emissions estimates is different for the two datasets. Both the Global EO and the Indonesian NGHGI applied IPCC Tier 1-style methodology, partially explaining the observed similarity in the gross removals component. Unfortunately, we could not attribute the similarities between the datasets in great detail given the lack of transparency and detail in the methodology and reporting of the NGHGI. For example, additional information is needed on the removal factors applied to the natural forests (old-growth or secondary forests). We compared the area of land type and found some differences between the two datasets; the area of natural forest cover in 2020 (old-growth and secondary forests) in the Global EO dataset is a third larger than the natural forest area in 2020 reported by Indonesia [52] (Table 7), suggesting there are differences in the definition of forest and deforestation. However, the ratio of swamp (peatland) forested areas to dry forested areas is similar between the NGHGI (0.15) and the Global EO (0.13) datasets (Table 7). The emission factors for primary forest carbon are 11 to 27% higher in the Global EO dataset than in the Indonesian NGHGI [53], potentially explaining some of the greater deforestation emissions in the Global EO dataset (Table 8).

**Table 7:** Area of forested and non-forest lands in Indonesia and Malaysia estimated by different flux datasets

Land type	Global EO (1000 ha)	NGHGI (1000 ha)
Indonesia		
Forested lands	Primary dry forest: 72,109.1 Primary swamp forest: 9017.7 Old secondary dry forest (> 20 yrs): 33,168.4 Old secondary swamp forest (> 20 yrs): 5220.0 **“Forest land remaining forest land” (primary + old secondary) subtotal: 119,515.1** Young secondary dry forest: 1772.8 Young secondary swamp forest: 197.5 **Natural forest subtotal: 121,485.5**	Primary dry forest: 41,029 Primary swamp forest: 4852 Secondary dry forest: 36,469 Secondary swamp forest: 6924 **Natural forest subtotal: 89,274**
Plantations	Forest plantation: 358.7 Tree plantation: 26,127.6 **Subtotal: 26,486.3**	Forest plantation: 5551 Tree plantation: 20,564 **Subtotal: 26,115**
Non-forested land	Other land: 40,890.0	Other land: 76,809
Total land area**	**188,861.8**	**192,198**
Malaysia		
Forested lands	Old-growth forest: 12,4483.4 Old secondary forest (> 20 yr): 4595.9 Young secondary forest: 361.9 **“Forest land remaining forest land” (old-growth + old secondary) subtotal: 17,079.3** **Natural forest subtotal: 17,441.2**	Forest land remaining forest land: 17,735.3
Plantations	Forest plantation: 0.0 Tree plantation: 10,613.8	*Cropland remaining cropland: 7037.9 *Forest land converted to cropland: 0.85 **Cropland total: 7038.8**
Non-forested land	Other land: 4922.5	Settlement remaining settlement: 2,327.1 Forest land converted to settlement: 137.1 Cropland converted to settlement: 21.9 **Settlement total: 2486.0** Grassland remaining grassland: 313.0
Total land area**	**32,977.5**	**27,573.2**

The datasets are the Global Earth Observation (EO) dataset and the National Greenhouse Gas Inventories (NGHGI). The data for the Global EO are for the year 2020 [17], for the Indonesian NGHGI for 2020 [49], and for the Malaysian NGHGI for 2019 [50]. The years for the NGHGI are the most up to data values as submitted to the UNFCCC. Units are in 1000 ha. The Indonesian NGHGI data includes data on the so-called “Area Penggunaan Lain”—APL areas which are largely considered non-forested but include some forested lands

Bold text denotes the subtotals/totals for each land type in the respective datasets and countries

^*^For the purpose of this study all croplands are assumed to be tree plantations

^**^Mangroves have been excluded from all datasets

**Table 8:** Emission and removal factors in Indonesia and Malaysia as estimated by different flux datasets

Emission factor type	Global EO (Mg C ha^−1^ yr^−1^)	NGHGI (Mg C ha^−1^ yr^−1^)
Indonesia		
Primary forest emission factor	Country average: 197.7	Dryland:176.6* Swamp forest: 142.7*
Malaysia		
Primary forest emission factor	Country average: 159.3	Inland State land forest: 140 PRF inland: 194
Removal factors	Old growth: 0.41 Old secondary forest (> 20 yrs): 1.60 Young secondary forest (< 20 yrs): 4.2 (min:1.3’; max: 10.6) Plantation forest: 3.0 to 14.1 Rubber: 3.4 Oil Palm: 3.02	Inland forest: 4.37 Peat swamp: 4.32 State land: 2.02 Plantation forest: 2.44 Rubber: 1.95 Oil Palm: 1.84

The data for the Global EO are for the year 2020 [17], for the Indonesian NGHGI for 2020 [49], and for the Malaysian NGHGI for 2019 [50]

^*^These values were presented in biomass and converted to carbon using the conversion factor of 0.47

**Fig. 9:**
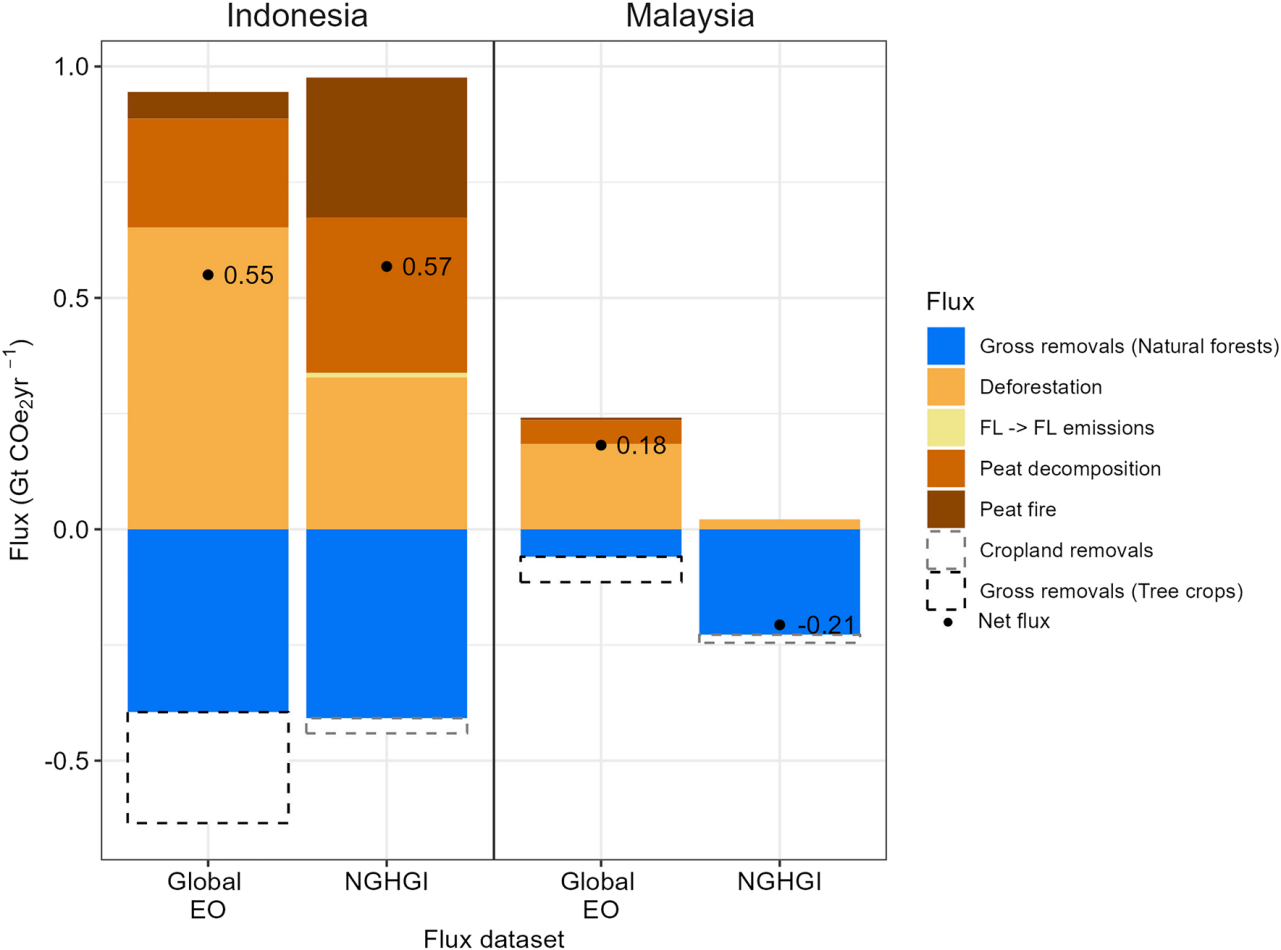
Average annual forest flux estimates in Indonesia and Malaysia according to different flux datasets. Bars denote the average annual gross emissions/removals and black points and associated text denote the net forest carbon fluxes over the period 2001 to 2020. The datasets are the Global Earth Observation (EO) dataset and the National Greenhouse Gas inventory (NGHGI). The raw data for the Global EO are available for 2001–2020 [17], for the Indonesian NGHGI for 2001–2019 [49], and for the Malaysian NGHGI for 2002–2016 [50]. For both the NGHGIs adjustments were made to the time period to make them comparable with the Global EO dataset (see “Methodology”). Gross emissions were separated according to the NGHGI categories, namely deforestation, emissions in forest land remaining forest land (FL → FL) (relating to biomass burning), peat decomposition, and peat fire removals. Gross removals refer to all-natural forest removals considered in the respective datasets. Gross removals in tree crop plantations in the Global EO were excluded as these are considered croplands by both NGHGIs. The net flux is shown by the black dot and associated number. Flux is given in CO_2_ equivalent (CO_2_e) as the Indonesian NGHGI did not subcategorise by gas, this distinction is only relevant for gross emissions and all gross removals are CO_2_-only

For Malaysia, we found that the estimated net fluxes from the NGHGI (− 0.2 GtCO2e yr^−1^) and Global EO (0.2 GtCO2e yr^−1^) were not only different in the magnitude (size) but also the sign (source or sink), despite a similar area of ‘forest land remaining forest land’ identified by both datasets (Table 7). One reason for the difference between the gross removal estimates in Malaysia may be linked to the high removal factor applied to all forest lands remaining forest lands by the NGHGI. For example, a removal factor of 4.37 MgC ha^−1^ yr^−1^ is applied to inland forests, and no distinction between secondary and old-growth forests is made (Table 8) [50]. The intact forest removal factor is about eleven and three times higher than the IPCC default values for old-growth and old secondary Asian tropical rainforests, respectively (Table 8) [54] and helps to explain the fourfold difference in the gross removals between the two datasets (Fig. 9). The intact forest value used by the NGHGI is more representative of young secondary forest regrowth rates in this region (Table 8) [35, 54]. The estimated gross removals flux of the NGHGI is, therefore, implausible [10].

The large difference between the gross emissions in Malaysia may be linked to the fact that most reported emissions and area change (> 99%) in the Malaysian NGHGI are associated with “forest land converted to settlement” (Table 7, Additional file 1: Tables S4 and S8). Conversely, the Global EO finds area change and associated emissions are dominated by “forest land converted to cropland”, with commodity driven agriculture making up 90% of gross emissions between 2001–2020 [55]. In 2019, the Global EO dataset estimates that approximately 373 kha of forest loss was commodity driven deforestation, a stark contrast to the 0.85 kha “forest land converted to cropland” as estimate d by the NGHGI for 2019. It is therefore surprising that the Malaysian NGHGI suggests practically no emissions from other land transitions, and reports only small net emissions from forest land converted to cropland (0.1MtCO_2_) in 2019 [50]. Finally, there also appears to be a difference between the total land area of Malaysia as estimated by the NGHGI (27,573 kha) and the Global EO (32,978 kha) (Table 7). This discrepancy of 18% may help to explain some of the observed difference in the total cropland area (3575 kha) reported and thus associated emissions resulting from forest land converted to cropland.

One potential reason for the difference in total area may be linked to the fact that the Malaysian NGHGI considers new forest area in State land to be in transition and therefore ‘not accounted for’ [50] (page 50). Similarly, for their Forest Reference Level (FRL) a small area of mountain and limited-access forests with no human induced activities are classified as unmanaged forest (0.925 Mha in 2015) [56]. Given that the Malaysian FRL is consistent with BUR4, such an assumption may also have been applied within the BUR4. In our study, we assumed all lands within the country boundary to be managed, and extracted Global EO data accordingly. The differences in land cover areas may explain some, but certainly not all of the observed differences in emissions and removals. It was not possible to do a more detailed geospatial, pixel-to-pixel, comparison of how each dataset classifies the data spatially, to explore the above hypotheses further.

## Discussion

We frame the following discussion around the five UNFCCC reporting principles included in the IPCC Guidelines, which are crucial pillars moving forward in Paris Agreement reporting [57]. The principles are: Comparability, Transparency, Consistency, Accuracy and Completeness. We discuss these principles in a broader sense, to how they were originally defined, to evaluate how they can help to understand and reconcile the differences between EO and national GHG inventories.

### Comparability: aligning definitions of “managed” forest

Our analysis highlights the influence of the managed land definition on estimates of gross emissions and removals from forest related fluxes (Fig. 2). Brazil’s NGHGI considered a large area of conservation and indigenous lands to be under anthropogenic influence and, therefore, “managed” (Fig. 3b and c). These areas are typically not highly degraded on scales detectable by moderate resolution satellite data such as Landsat, and so were classified as old-growth (primary) forests by the dataset used by the Global EO method used in this analysis [17, 45]. Using a global mask of non-primary forest to represent managed land was, therefore, not the best approach to accurately align the Global EO data with the NGHGI in Brazil [7]. A previous study reconciling country-scale NGHGIs and Bookkeeping models found similar results when using a global intact-forest mask in Brazil [13]. For Brazil, it is possible to directly apply the same mask of Managed Land as considered by the NGHGI to the Global EO, as the spatially explicit dataset of LULC in the NGHGI is made public.

In our study, a key source for the differences between the estimates of the flux datasets was the extent to which different forest categories were included in relation to the IPCC categories (Table 2). Given the differences in scope, purpose, and capacity of the respective flux datasets, each included the various types of forest transitions in unique ways. High granularity of the Global EO data, in terms of its components and spatial data, gave it flexibility such that adjustments could be made to reflect NGHGIs approaches and definitions, thus making it a useful tool for MRV purposes. Interpretation and definitions of forest type vary between approaches and datasets [58]. The category “old secondary forest” in the Global EO flux dataset was most akin to “secondary forest remaining secondary forest” in the Brazilian NGHGI and was removed in adjustments (Fig. 6), as this category is not considered a flux source by Brazil’s NGHGI (Table 2). A future improvement to the Brazilian NGHGI, may be to include GHG fluxes in all sub-categories of managed forests, and not only for old-growth managed forest and land converted to forest. For example, including GHG fluxes from secondary forests remaining secondary forests would provide a more complete representation of forest-related fluxes, with the additional benefit of also quantifying the effectiveness of GHG mitigation measures.

While the vast majority of countries report some GHG fluxes from land, only Annex I countries plus a few Non-Annex I countries explicitly report the areas of managed land associated with these GHG fluxes and the area of unmanaged land for which no GHG flux is reported [10]. For most Non-Annex I countries, the area of managed land associated with the reported GHG fluxes, and any possible area of unmanaged land, remain unclear or implicit in the NGHGI [10]. Under the ambition to improve inventories, Malaysia, Indonesia, and other countries in a similar situation, may consider explicitly defining unmanaged lands separately from managed lands. With the Paris Agreement and Enhanced Transparency Framework, countries will have to fill in new, common reporting table of GHG fluxes that will naturally bring increased clarity on how countries separate managed vs unmanaged lands. Additionally, there is scope for countries that have not provided any information so far to consider the advantages and disadvantages of explicitly defining all their land as managed [59, 60]. Reporting all emissions and removals would provide a complete picture of the fluxes occurring on the land [59] and be more directly comparable with other NGHGIs, and with other independent flux datasets, such as the Global EO dataset used in this study. Alternatively, for lands explicitly considered unmanaged, providing flux data for these regions could be used for information purposes only, and would not necessarily account towards the countries’ climate targets. In any case, separately reporting emissions and removals in different forest types (e.g. primary forest, secondary forest etc.) would increase comparability as well as transparency.

### Transparency and consistency: methods, definitions, and data sources

Prior to the Paris Agreement, non-Annex 1 countries had met their responsibilities on the level of reporting required within the Kyoto Protocol. Common, but differentiated responsibilities recognised that non-Annex 1 countries (a) had limited capacity and (b) had less historical responsibility in terms of emissions contribution. Under the Enhanced Transparency Framework, there is now greater requirement for transparency and all countries will be required to provide a Biennial Transparency Report (BTR) from 2024 [47] to improve consistency and transparency among all NGHGIs [60]. The BTP will also help to ensure consistency between submissions, helping the task of the inventory reviewer. Furthermore, there is an increasing call for the research community to contribute more to emerging MRV needs under the Paris Agreement [61, 62]. For this engagement to be effective, transparent reporting and freely available methods and datasets are required by the research and the national inventory communities [58].

Transparent methods used in each of Brazil’s datasets made it possible to distinguish and quantify methodological and definitional differences such as forest categories. This transparent information enabled adjustments to be made to improve comparability and thus credibility of the estimates. Unfortunately, such a geospatial analysis was not possible in other countries, such as Indonesia and Malaysia, where digitised versions of the LULC maps were unavailable for open-access analysis. While it may not always be possible to fully reconcile differences between datasets as each has their own motivations and assumptions, the differences should be traceable so they can be understood. For greater transparency, countries could consider clearly documenting the emissions and removal factors used in their submissions to the UNFCCC, as was demonstrated in the Malaysian BUR4.

All the NGHGIs considered in our study clearly stated that tree crops such as palm oil and rubber are classified as perennial agricultural crops. Associated emissions and removals are, therefore, not necessarily forest-related (Tables 1, 2). The Global EO included tree crops broadly within their definition of plantations (Tables 1, 2). In this case, high transparency in the definitions used by each NGHGI enabled appropriate adjustments to be made to the EO dataset for a suitable comparison, demonstrating the value of disaggregated and flexible geospatial data.

Variations in definitions are not limited to our analysis and vary between global models and other internationally applied flux datasets such as the FAO [10], emphasising the need for transparency in definitions and delineating which IPCC land use categories are included. Future updates to the Global EO flux dataset [11] could include spatial disaggregation of the fluxes following the land use category definitions and the different forest types used in countries’ GHG inventories. Newer versions of the Global EO dataset now provide annual emissions estimates, this higher temporal granularity could be expanded to the gross removal estimates. This would improve the consistency and enable a more representative comparison with NGHGIs.

### Accuracy and completeness: differences in forest types and removal factors—expanding definitions

For completeness, there is a need to consider all forest types, which might be GHG sources or sinks. EO data can help capture spatial and temporal heterogeneity of forest types and associated fluxes, to improve accuracy.

Our analysis has shown spatial differences in the forest cover between the Brazilian NGHGI and Global EO approach, especially outside the Amazonia biome, with divergences in the savannah and dry forest biomes (Fig. 5). These discrepancies explain some of the differences between the fluxes (Fig. 2) and may be an important consideration for carrying out similar analysis elsewhere. In increasingly forest fragmented regions such as in the Cerrado, it is important to accurately distinguish forested areas from other naturally existing vegetation, such as other woody vegetation, shrublands and natural grasslands, to track transitions for a complete representation [16]. Such distinctions can be aided by visual interpretation and knowledge of the areas by experts consulted during preparation of the NGHGIs, thus highlighting the potential for NGHGIs to refine methods used by global EO datasets.

Across the Brazilian biomes, fire and logging disturbances can cause forest degradation that often occurs at smaller-scales, and thus goes undetected by moderate resolution (> 30 m) remote sensing products [15]. Due to its complexity, the impact of forest degradation on emissions, and subsequent recovery, is currently poorly constrained in the Global EO dataset, NGHGIs, and other global models [2, 17, 38, 43]. As a result, motivation in NDC pledges to tackle forest degradation has trailed behind limiting deforestation [63]. Individual studies focusing on forest degradation have quantified and shown that degradation is a sizeable contribution to forest carbon emissions [64, 65], but fewer studies have focused on the recovery of such degraded forests [66–68]. There is a need to expand the current definitions of forest types to accurately represent degraded forests, to avoid underestimating key sources of carbon emissions and miss potential opportunities to protect and increase the carbon sink in recovering degraded forests. Independent EO data may be a useful tool to provide spatial, highly granular information that is not currently considered by NGHGIs and fill information gaps, providing additional knowledge for previously unmonitored or unaccounted (mitigation) processes.

Our analysis highlighted the differences in the removal factors used by different datasets as an important discrepancy, demonstrating the ongoing uncertainty with regards to applying removal factors for different forest types. Per unit area, the difference was particularly evident in young secondary forests in Brazil (“other land converted to secondary forests”) (Table 6). However, the total removals associated with these young secondary forests are modest (Table 4), so the overall impact on the total gross removals is small. The difference between the old-growth forest removal factors was less, around ~ 25%, but given the large area extent of old-growth forest, the difference in the total removals was considerable (Table 5).

As many countries and organisations aim to reduce their net carbon emissions through forest conservation and restoration, accurate and representative removal factors for all forest types [42, 45, 56] are essential for credibility [54, 61, 62]. Inaccurate removal factors risk overestimating sinks and could lead to reduced ambition in reducing fossil fuel emissions. In the Malaysian NGHGI, we found the Tier-2 style removal factor used for inland “old-growth” forests to be more representative of young secondary forests, suggesting there may be inaccuracies that warrant further analysis. Remote sensing studies that analyse changes in AGC in space [37] and/or time [65] may provide useful information to improve the accuracy of removal factors thus providing Tier-2 or Tier-3 information which is crucial for countries in which LULUCF emissions are high, such as the ones analysed here. There is also scope to expand the approach applied here to encompass other EO datasets [16], and help to contextualise the approach used by the EO dataset in this study, which relied in part on using IPCC Tier-1 style removal factors [17]. With the increased expectations for countries’ NGHGI reporting, the IPCC inventory guidelines and their current process should not go unchallenged, with potential to also improve in terms of their accuracy, completeness and timeliness [69].

In this study, we highlighted the differences between the flux datasets regarding the forest classification assigned to a given pixel in space and time, which has implications for the emission/removal factor applied [26]. The impact of differences in forest type was most noticeable in the classification of “old secondary forest” in the Global EO dataset, of which many regions were classified as managed old-growth forests by the Brazilian NGHGI and SEEG (Fig. 7). Future work identifying forests according to the type [15, 34] and intensity of disturbance [70] may be a useful starting point for more disaggregated classification. Expanding the representation of forest demographics beyond simply < 20 and > 20 years would also enable more accurate removal factors to be applied. Where feasible, there may be scope to align the definitions of forest type used by different flux datasets. This would enable a more credible comparison and accurate assessment [58, 71]. Nevertheless, there are likely to be difficulties in negotiating definitions among different approaches and countries’ NGHGIs.

## Conclusions

Independent data, such as that from the Global EO model included here, have the potential to provide key sources of information for the MRV of NGHGIs [58]. Conversely, information from NGHGIs may be useful to refine the methods and data used in the Global EO approach, for example by using region-specific removal factors.

Using Brazil as a primary case study, we could reconcile the difference between GHG fluxes from a Global EO dataset and the NGHGI. With no adjustment to the Global EO dataset, considering all forest fluxes in the country boundary, the difference between the Global EO flux (− 0.2 GtCO_2_ yr^−1^) and the NGHGI flux (0.8 GtCO_2_ yr^−1^) from 2001 to 2020 was 1.0 GtCO_2_ yr^−1^ and estimates were opposite in sign. Adjusting the Global EO flux by applying the Brazilian NGHGI definition of managed forest area and only considering the assumptions in their inventory (in terms of land transition and flux categories) resulted in the adjusted Global EO net flux to be 0.6 GtCO_2_ yr^−1^, now just 0.2 GtCO_2_ yr^−1^ lower compared to the NGHGI. The various adjustments also highlight the impact of considering all land as managed versus different applications of the managed land proxy. The analysis was made possible due to the availability of the geospatial data and transparency in methodologies from all flux datasets.

In the other two case study countries, Indonesia and Malaysia, the analysis was limited to the tabular data provided by the respective NGHGIs as no spatially explicit datasets on forest cover types were publicly available. Limited transparency in the emission and removal factors used also restricted detailed analysis. However, a comparative analysis indicated where differences and uncertainties exist and where areas for improvement in both the NGHGIs and Global EO datasets can lead to increasing harmonisation.

The adjustments outlined in this study can also be used to aid comparisons with and benchmarking of other approaches, such as global models or in other regions. Below we outline some key lessons learned in this study:

- Effective comparisons require more clarity by countries in their use of the Managed Land Proxy and IPCC forest categories. Making the appropriate adjustments to account for differences between datasets becomes increasingly important when using independent flux datasets for MRV at (sub)-country scales. For completeness and information purposes, NGHGIs could voluntarily consider providing fluxes associated to unmanaged land or consider all land to be managed.
- Full transparency is crucial for understanding differences and making effective comparisons between datasets. This includes ensuring transparency in the methodology and open access to the data used by all approaches.
- Further disaggregation in the forest types and associated fluxes in all datasets will facilitate greater comparability and completeness. Independent datasets could disaggregate the fluxes by the land use categories and forest types used in NGHGIs, to allow greater comparability. All flux datasets could explicitly consider disturbance type and intensity, and associated recovery.

As more information and datasets become available, including EO, countries’ requirements and capacity to report is expected to increase in transparency, comparability, consistency, completeness, and accuracy. Additionally, region specific information compiled for use in NGHGIs can also be used to improve local applicability of Global EO assessments. Given the approval of Article 6.4 of the Paris Agreement, and the numerous pledges and commitments that have been made by business organisations, cities, nations, and international agreements to protect and restore forests, the need to measure, report and especially verify estimates becomes increasingly important. If this is not achieved in accurate and credible ways, we risk mis-representing the carbon fluxes of the world’s forests, one of our most important allies on land, to tackle the climate emergency.

### Supplementary Information

**Additional file 1: Figure S1.** Comparison of the area of Primary and non-Primaryforest land according to the Global EO (Harris et al. 2021) in the year 2000, for which they used dataset byTurubanova et al. (2018)and the Unmanaged forest land and Managed forest land according to the NGHGI in 1994 and 2002. **Figure S2.** Percentage contribution of different plantation types to the total plantation area in different biomes of Brazil. Colouring denotes if the plantation is a forest plantation (shades of blue) or a Tree crop plantation (yellow-oranges). Theareas considered for analysis only included those that were also classified as plantation area in the National Greenhouse gas inventory of Brazil in 2016. **Figure S3.** Bar graphs representing the area contribution of different forest and non-forest cover types that make up the six biomes of Brazil according to three different approachesfor the year 2020. The three approachesare the Global Earth Observation (EO), the National Greenhouse Gas Inventory (NGHGI) and the independent estimate (SEEG). As the NGHGI is only available up to 2016, area numbersfor theNGHGIhave been adjusted by multiplying by the fractional difference in the area of each forest type in 2016 and 2020 according to SEEG (Mapbiomas).Note differences in the Y axis scale. **Figure S4.** Bar graphs representing theaveragegross removals contribution of different forest types within the six biomes of Brazil according to three different approachesover the period 2001 to 2020.The three approachesare the Global Earth Observation (EO), the National Greenhouse Gas Inventory (NGHGI) and the independent estimate (SEEG). As the NGHGI is only available up to 2016, numbersfor the NGHGI have been adjusted by multiplying by the fractional difference in the removalsof each forest type in 2016 and 2020 according to SEEG.Note differences in the Y axis scale. **Table S1.** Summary of the key United Nations Framework Convention on Climate Change (UNFCCC) principles as outlined in the 5th Conference of the Parties (COP5) in 1999. **Table S2.** Modifications made to the NGHGI of Brazil to account for the shorter time-period available. The fractional difference of the average fluxes were calculated for the period 2002 to 2016 (period of the NGHGI) and for 2001 to 2020 (period of the Global EO). **Table S3.** The total number of pixels per biome which are considered as non-primary forests in the Global Earth Observation dataset and overlap pixels classified as Managed forests (old-growth managed, secondary forest, plantation forest) pixels in the National Greenhouse Gas Inventory (NGHGI) of Brazil. **Table S4.** The categories of forest-based transitions used within this study and how they are referred to in the National Greenhouse Gas Inventory (NGHGI) of the case study countries Indonesia and Malaysia.

## Data Availability

All data used in this research is available from the sources that are fully referenced in the text and bibliography. CO_2_-only fluxes from the Global EO dataset are readily available from the WRI upon request. The processed data can be downloaded here: https://zenodo.org/records/10043625.
