# Supplementary material for: Mind the gap: reconciling tropical forest carbon flux estimates from earth observation and national reporting requires transparency
Source: Carbon Balance Manag. 2023 Nov 20;18:22. doi: 10.1186/s13021-023-00240-2 (PMC10662451; doi:10.1186/s13021-023-00240-2)
Supplement: Supplementary file 1 — Additional file 1: Figure S1. Comparison of the area of Primary and non-Primaryforest land according to the Global EO (Harris et al. 2021) in the year 2000, for which they used dataset byTurubanova et al. (2018)and the Unmanaged forest land and Managed forest land according to the NGHGI in 1994 and 2002. Figure S2. Percentage contribution of different plantation types to the total plantation area in different biomes of Brazil. Colouring denotes if the plantation is a forest plantation (shades of blue) or a Tree crop plantation (yellow-oranges). Theareas considered for analysis only included those that were also classified as plantation area in the National Greenhouse gas inventory of Brazil in 2016. Figure S3. Bar graphs representing the area contribution of different forest and non-forest cover types that make up the six biomes of Brazil according to three different approachesfor the year 2020. The three approachesare the Global Earth Observation (EO), the National Greenhouse Gas Inventory (NGHGI) and the independent estimate (SEEG). As the NGHGI is only available up to 2016, area numbersfor theNGHGIhave been adjusted by multiplying by the fractional difference in the area of each forest type in 2016 and 2020 according to SEEG (Mapbiomas).Note differences in the Y axis scale. Figure S4. Bar graphs representing theaveragegross removals contribution of different forest types within the six biomes of Brazil according to three different approachesover the period 2001 to 2020.The three approachesare the Global Earth Observation (EO), the National Greenhouse Gas Inventory (NGHGI) and the independent estimate (SEEG). As the NGHGI is only available up to 2016, numbersfor the NGHGI have been adjusted by multiplying by the fractional difference in the removalsof each forest type in 2016 and 2020 according to SEEG.Note differences in the Y axis scale. Table S1. Summary of the key United Nations Framework Convention on Climate Change (UNFCCC) principles as outlined in the 5th [file 13021_2023_240_MOESM1_ESM.docx]

Supplementary Information for:

# Mind the Gap: Reconciling tropical forest carbon flux estimates from Earth Observation and National Reporting requires transparency

**Authors**

Viola H. A. Heinrich^1,2,3*^,Jo House^1^, David A. Gibbs^4^, Nancy Harris^4^, Martin Herold^3,5^, Giacomo Grassi^6^, Roberta Cantinho^7^, Thais M. Rosan^2^, Barbara Zimbres^8^, Julia Z. Shimbo^8^, Joana Melo^6^, Tristram Hales^9^, Stephen Sitch^2^, Luiz E. O. C. Aragão^2,10^

**Affiliations and Addresses**

^1^School of Geographical Sciences, University of Bristol, Bristol, UK.

^2^Faculty of Environment, Science and Economy, University of Exeter, Exeter, UK.

^3^Helmholtz GFZ German Research Centre of Geosciences, Section 1.4 Remote Sensing and Geoinformatics, Telegrafenberg, Potsdam, Germany

^4^World Resources Institute, Washington DC, USA

^5^Wageningen University and Research, Wageningen, the Netherlands.

^6^Joint Research Centre, European Commission, Ispra, Italy.

^7^Centre for Sustainable Development (CDS), University of Brasília (UnB), Brasília, Brazil.

^8^Amazon Environmental Research Institute (IPAM), Brasília, Brazil.

^9^School of Earth and Environmental Sciences, Cardiff University , Cardiff, UK.

^10^Earth Observation and Geoinformatics Division, National Institute for Space Research (INPE), São José dos Campos, Brazil.

*Corresponding author:

Email: [viola.heinrich@gfz-potsdam.de](mailto:viola.heinrich@gfz-potsdam.de)

**Supplementary Figures**

**Supplementary Figure S1** Comparison of the area of Primary and non-Primary forest land according to the Global EO (Harris et al. 2021) in the year 2000, for which they used dataset by Turubanova et al. (2018) and the Unmanaged forest land and Managed forest land according to the NGHGI in 1994 and 2002.


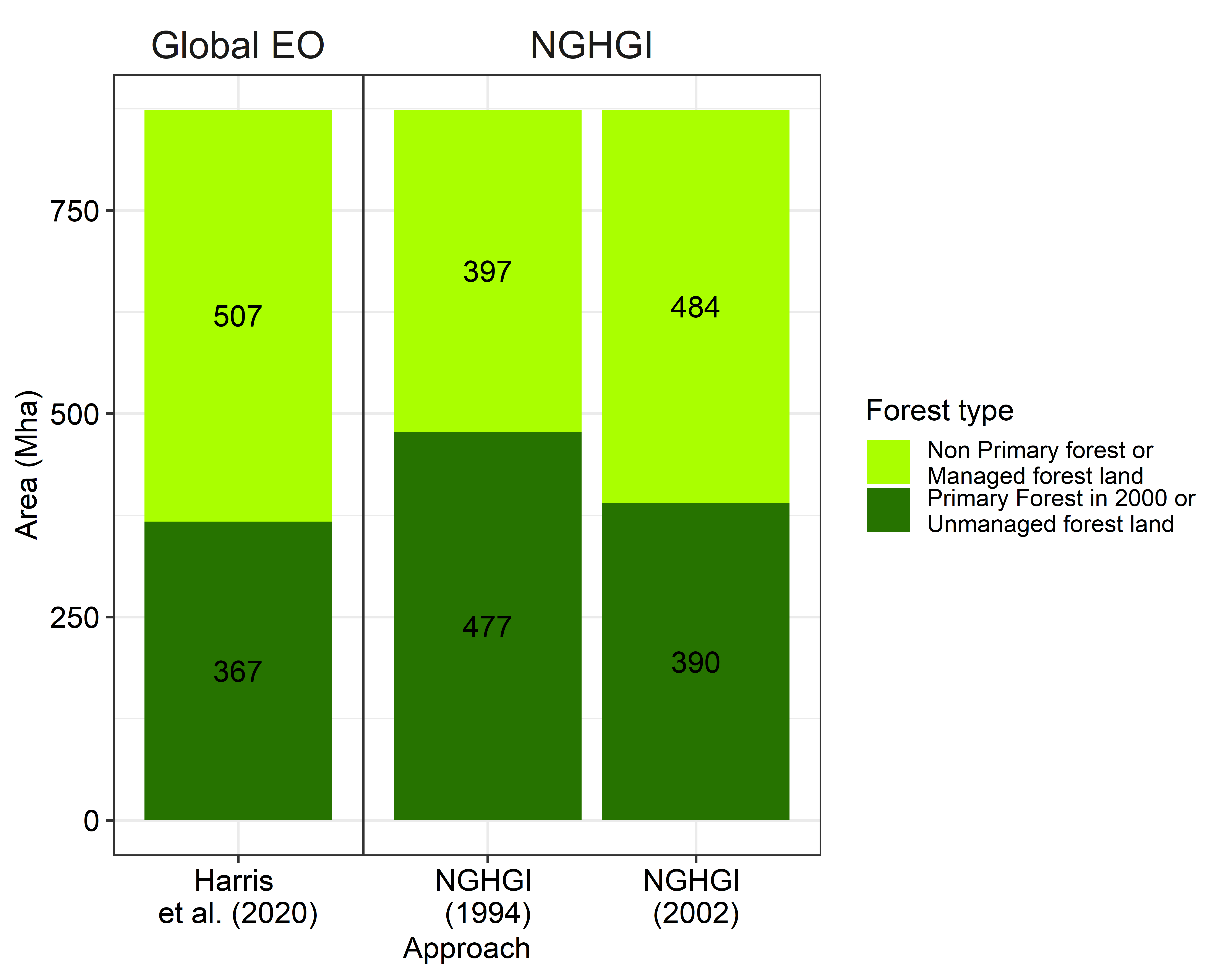

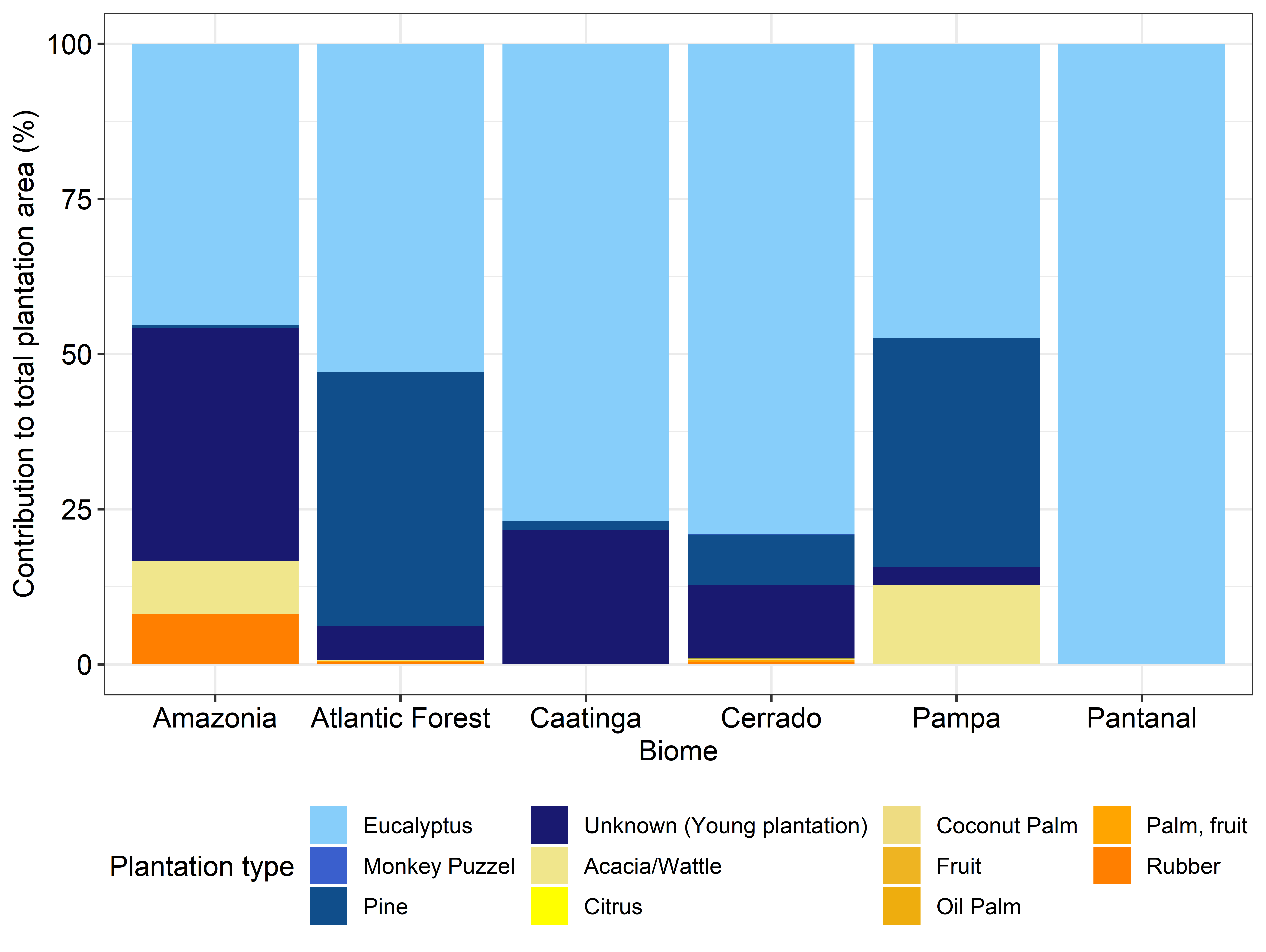


**Supplementary Figure S2** Percentage contribution of different plantation types to the total plantation area in different biomes of Brazil. Colouring denotes if the plantation is a forest plantation (shades of blue) or a Tree crop plantation (yellow-oranges). The areas considered for analysis only included those that were also classified as plantation area in the National Greenhouse gas inventory of Brazil in 2016.

**Supplementary Figure S3** Bar graphs representing the area contribution of different forest and non-forest cover types that make up the six biomes of Brazil according to three different approaches for the year 2020. The three approaches are the Global Earth Observation (EO), the National Greenhouse Gas Inventory (NGHGI) and the independent estimate (SEEG). As the NGHGI is only available up to 2016, area numbers for the NGHGI have been adjusted by multiplying by the fractional difference in the area of each forest type in 2016 and 2020 according to SEEG (Mapbiomas). Note differences in the Y axis scale.


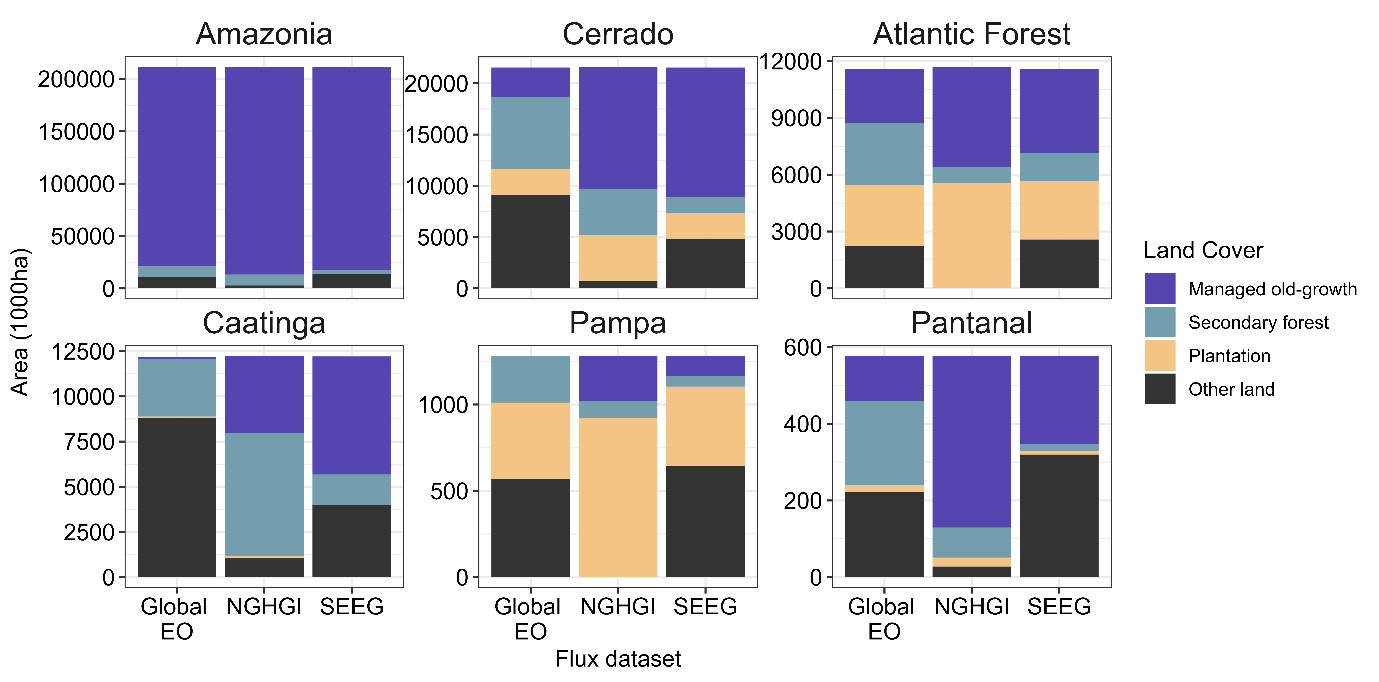


**Supplementary Figure S4** Bar graphs representing the average gross removals contribution of different forest types within the six biomes of Brazil according to three different approaches over the period 2001 to 2020. The three approaches are the Global Earth Observation (EO), the National Greenhouse Gas Inventory (NGHGI) and the independent estimate (SEEG). As the NGHGI is only available up to 2016, numbers for the NGHGI have been adjusted by multiplying by the fractional difference in the removals of each forest type in 2016 and 2020 according to SEEG. Note differences in the Y axis scale.


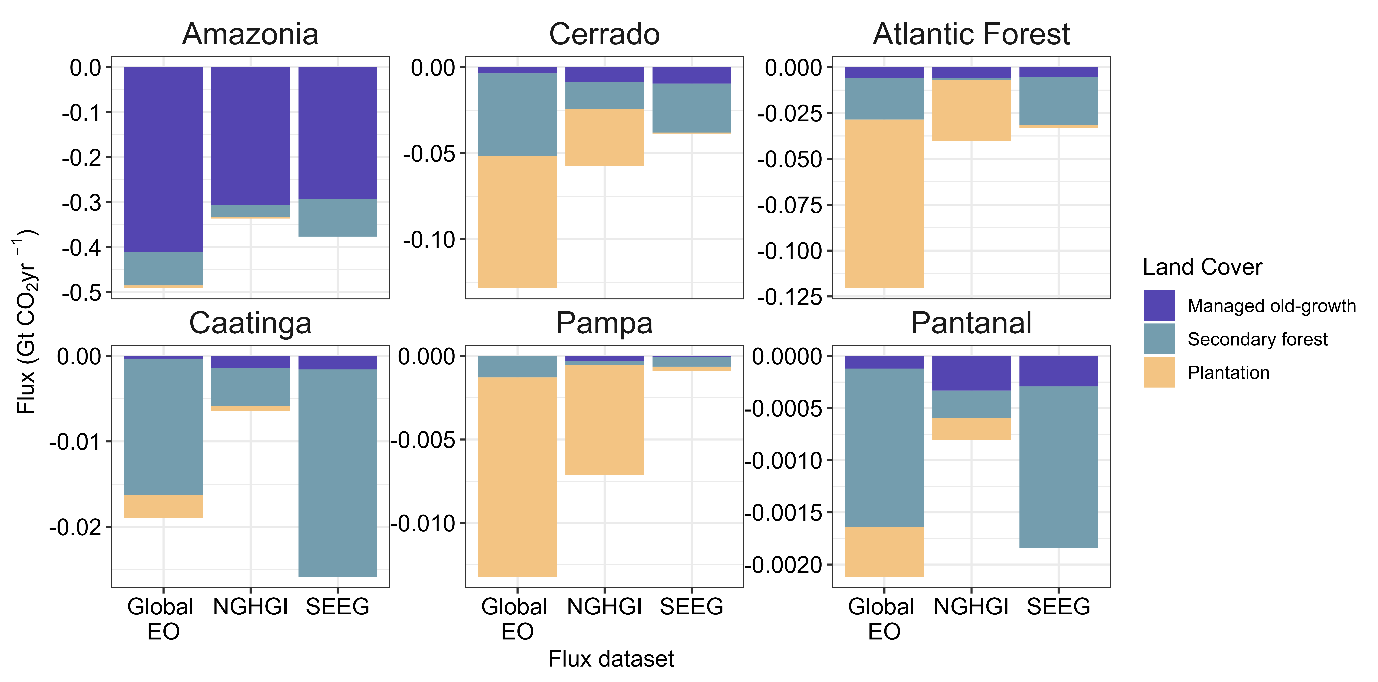


**Supplementary Tables**

**Supplementary Table S1.** Summary of the key United Nations Framework Convention on Climate Change (UNFCCC) principles as outlined in the 5^th^ Conference of the Parties (COP5) in 1999. ^1^

| **UNFCCC principle** | **Definition** |
| --- | --- |
| Transparency | “…assumptions and methodologies used… should be clearly explained to facilitate replication and assessment…” |
| Consistency | “…inventory should be internally consistent in all its elements with inventories of other years” |
| Comparability | “…emission and removals reported by Parties in inventories should be comparable among Parties…” |
| Completeness | “… an inventory should cover all sources and sinks, as well as all gases...” |
| Accuracy | “… a relative measure of the exactness of an emission or removal estimate…” |

**Supplementary Table S2.** Modifications made to the NGHGI of Brazil to account for the shorter time-period available. The fractional difference of the average fluxes were calculated for the period 2002 to 2016 (period of the NGHGI) and for 2001 to 2020 (period of the Global EO.

| **Flux** | **2002 to 2016 average**  **(GtCO_2_ yr^-1^)** | | **Fractional difference** | **2001 to 2020 average (GtCO_2_ yr^-1^)** | **Fractional adjustment 2001 to 2020 (GtCO_2_ yr^-1^)** |
| --- | --- | --- | --- | --- | --- |
|  | SEEG | NGHGI | SEEG÷NGHGI | SEEG | NGHGI |
| **Gross emissions** | 1.08 | 1.32 | 0.96 | 1.04 | 1.27 |
| **Gross removals** | -0.46 | -0.44 | 1.03 | -0.48 | -0.45 |
| **Net flux** | 0.62 | 0.89 | 0.9 | 0.56 | 0.80 |

**Supplementary Table S3.** The total number of pixels per biome which are considered as non-primary forests in the Global Earth Observation dataset and overlap pixels classified as Managed forests (old-growth managed, secondary forest, plantation forest) pixels in the National Greenhouse Gas Inventory (NGHGI) of Brazil

| **Brazilian Biomes** | **Number of non-primary forest pixels overlapping NGHGI Managed Forest pixels** | **Total number of non-primary forest pixels** | **Percentage of non-primary forest pixels overlapping NGHGI Managed Forest pixels (%)** |
| --- | --- | --- | --- |
| **Amazonia** | 1.91E+08 | 1.06E+09 | 18.10 |
| **Cerrado** | 2.13E+08 | 2.25E+09 | 9.43 |
| **Atlantic Forest** | 1.05E+08 | 1.28E+09 | 8.21 |
| **Caatinga** | 1.37E+08 | 9.33E+08 | 14.68 |
| **Pampa** | 1.66E+07 | 2.14E+08 | 7.74 |
| **Pantanal** | 5160270 | 1.59E+08 | 3.25 |
| **TOTAL** | **6.68E+08** | **5.90E+09** | **11.33** |

**Supplementary Table S4.** The categories of forest-based transitions used within this study and how they are referred to in the National Greenhouse Gas Inventory (NGHGI) of the case study countries Indonesia and Malaysia.

| **Country** | **Category used within this study as called within respective NGHGIs** |
| --- | --- |
| Indonesia | **Gross removals (Natural forests):**  Forest land remaining forest, Non-forest to forest land  **FL -> FL emissions:**  Biomass burning  **Deforestation:**  Non-forest to forest, Non-cropland to cropland, Non-settlement to settlement, Non-other land to other land, Peat Decomposition  **Peat fire:**  Peat Fire  **Cropland removals:**  Cropland remaining cropland (shown in analysis but not included in calculations) |
| Malaysia  (see page 188 onwards of BUR4) | **Gross removals (Natural forests):**  Forest land (a net flux rather than gross removals)  **Deforestation:**  Settlement (assumed all emissions are from deforestation)  Biomass burning  Managed soils emissions  **Cropland:**  Cropland (shown in analysis but not included in calculations) |
